# Engineering a genome‐reduced bacterium to eliminate *Staphylococcus aureus* biofilms *in vivo*

**DOI:** 10.15252/msb.202010145

**Published:** 2021-10-06

**Authors:** Victoria Garrido, Carlos Piñero‐Lambea, Irene Rodriguez‐Arce, Bernhard Paetzold, Tony Ferrar, Marc Weber, Eva Garcia‐Ramallo, Carolina Gallo, María Collantes, Iván Peñuelas, Luis Serrano, María‐Jesús Grilló, María Lluch‐Senar

**Affiliations:** ^1^ Centre for Genomic Regulation (CRG) The Barcelona Institute of Science and Technology Barcelona Spain; ^2^ Institute of Agrobiotechnology (IdAB; CSIC‐Navarra Government) Mutilva Spain; ^3^ Pulmobiotics Ltd Barcelona Spain; ^4^ S‐Biomedic N.V. Beerse Belgium; ^5^ RADIOMIN Research Group Clínica Universidad de Navarra Pamplona Spain; ^6^ IdiSNA, Navarra Institute for Health Research Pamplona Spain; ^7^ Universitat Pompeu Fabra (UPF) Barcelona Spain; ^8^ ICREA Barcelona Spain; ^9^ Basic Sciences Department Faculty of Medicine and Health Sciences Universitat Internacional de Catalunya Sant Cugat del Vallès Spain

**Keywords:** bacterial therapy, biofilm, *in vivo*, *Mycoplasma*, synthetic biology, Biotechnology & Synthetic Biology, Microbiology, Virology & Host Pathogen Interaction

## Abstract

Bacteria present a promising delivery system for treating human diseases. Here, we engineered the genome‐reduced human lung pathogen *Mycoplasma pneumoniae* as a live biotherapeutic to treat biofilm‐associated bacterial infections. This strain has a unique genetic code, which hinders gene transfer to most other bacterial genera, and it lacks a cell wall, which allows it to express proteins that target peptidoglycans of pathogenic bacteria. We first determined that removal of the pathogenic factors fully attenuated the chassis strain *in vivo*. We then designed synthetic promoters and identified an endogenous peptide signal sequence that, when fused to heterologous proteins, promotes efficient secretion. Based on this, we equipped the chassis strain with a genetic platform designed to secrete antibiofilm and bactericidal enzymes, resulting in a strain capable of dissolving *Staphylococcus aureus* biofilms preformed on catheters *in vitro*, *ex vivo*, and *in vivo*. To our knowledge, this is the first engineered genome‐reduced bacterium that can fight against clinically relevant biofilm‐associated bacterial infections.

## Graphical abstract

A non‐pathogenic strain of *Mycoplasma pneumoniae* is engineered to express biofilm dispersing agents as well as bactericidal peptides against *Staphylococcus aureus*. The engineered strain efficiently dissolves *S. aureus* biofilms *in vitro* and *in vivo*.

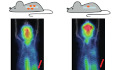

## Introduction

The use of genetically programmed microorganisms opens the door to alternative therapies based on a continuous or regulated targeted release of therapeutic molecules in a desired location (Piñero‐Lambea *et al*, 2015). In the past years, many of the new drugs used in the clinics have been biomolecules, such as antibodies, interleukins, and enzymes, which are often administered systemically (Valeur *et al*, 2017). Production of these biomolecules is generally expensive, and the requirement for systemic administration in some cases prevents their use due to toxicity. As an alternative, local production of these biomolecules by a living system (i.e., bacteria) represents an attractive approach to not only reduce the production costs but also potential undesired effects associated with systemic administration.

There are already bacterial therapies in different phases of development against a wide variety of diseases, such as cancer (Duong *et al*, 2019), metabolic diseases (Isabella *et al*, 2018; Kurtz *et al*, 2019), viral infections (Lagenaur *et al*, 2011; Álvarez *et al*, 2015), and autoimmune disorders (Shigemori & Shimosato, 2017; Praveschotinunt *et al*, 2019). Noticeably, there are also therapeutic strains that have been programmed to destroy other bacteria (Hwang *et al*, 2018), taking advantage of the mechanisms by which bacteria compete with each other in nature (Granato *et al*, 2019).

Although the field of bacterial therapy is unarguably growing, most of the examples mentioned above are based on a handful of well‐known bacterial genera, such as *Escherichia* or *Lactococcus*. This is due in some cases to the lack of genome editing tools for other bacteria. Fortunately, our ability to edit bacterial genomes is increasing even to the most genetically intractable genera (Krishnamurthy *et al*, 2016). This opens the possibility of adapting other strains as therapeutic vectors that might be more suitable for a particular organ and disease than the traditional “lab work horses” (Adams, 2016).

We have chosen to use the genome‐reduced bacterium *Mycoplasma pneumoniae* as a potential new therapeutic delivery vector. This streamlined genome (816 kbp) (Himmelreich *et al*, 1996) has a set of features of interest for a bacterial‐based therapeutic vector: (i) It is one of the bacteria for which more quantitative and extensive datasets is available (Güell *et al*, 2009; Kühner *et al*, 2009; Yus *et al*, 2009; Wodke *et al*, 2013; Lluch‐Senar *et al*, 2015); (ii) it has simplified metabolic and gene networks, thereby reducing the risk of interference with *de novo* programmed abilities; (iii) it has intrinsic containment measures for horizontal gene transfer (HGT). For instance, it uses an UGA triplet as a tryptophan codon rather than a stop codon (Osawa *et al*, 1990), and it has a weakened recombination capacity (Krishnakumar *et al*, 2010; Sluijter *et al*, 2010, 2012); these traits limit both the acquisition and the release of DNA‐encoded information; and iv) it does not have a cell wall. The lack of a cell wall is important for several reasons: It is likely to lessen detection by the host immune system, as cell wall components are generally major targets of immune system recognition (Sukhithasri *et al*, 2013); it facilitates the direct release of secreted bioactive compounds; and it provides the possibility of targeting the cell wall of a pathogenic bacteria by using a combination of antibiotics together with a mycoplasma‐based therapeutic vector.

*M. pneumoniae* is generally considered to be a mildly infectious agent, and its few pathogenic determinants are well characterized (He *et al*, 2016). Specifically, *M. pneumoniae* infection starts by initially adhering to the sialoglycoproteins and sulfated glycoproteins exposed on the surface of the respiratory epithelia (Razin, 1999; Chaudhry *et al*, 2007). Adhesion is mediated by a specialized organelle composed of a main adhesin located at tip of this structure (i.e., P1 adhesin) and up to 7 different accessory proteins and adhesins (i.e., HMW1‐3 polypeptides, and the P30, P40, P90, and P65 adhesins) (Chaudhry *et al*, 2007). Disruption of proteins involved in the organelle results in non‐pathogenic and non‐adherent strains (Kahane, 1984; Romero‐Arroyo *et al*, 1999). The membrane‐associated nuclease, encoded by *mpn133*, is internalized by human cells and shows a cytotoxic activity (Somarajan *et al*, 2010). In addition, the cytopathic effect of *M. pneumoniae* can be explained by the production of hydrogen peroxide as a metabolic subproduct of GlpD activity, an enzyme encoded by *mpn051* gene that is involved in glycerol assimilation (Hames *et al*, 2009). Lastly, the pathogenesis of *M. pneumoniae* is also due to the production of a dedicated toxin, termed community‐acquired respiratory distress syndrome (CARDS) toxin, encoded by the *mpn372* gene (Kannan & Baseman, 2006). This toxin is internalized via clathrin‐mediated endocytosis (Krishnan *et al*, 2013), catalyzes adenosine diphosphate ribosylation (ADP‐ribosylation), and induces a highly vacuolated phenotype in the host cells (Kannan & Baseman, 2006). Removal of these pathogenic gene determinants can now be done systematically given the recent development of genome editing tools for this bacterium (Piñero Lambea *et al*, 2020).

One of the most relevant agents causing insidious clinical infections is *Staphylococcus aureus* (Tong *et al*, 2015). Biofilms formed by *S. aureus* are a major problem in hospital settings, in particular in patients with indwelling medical devices (Moormeier & Bayles, 2017). Biofilms are multi‐layered organized structures composed of bacterial cells embedded in an extracellular polymeric substance (EPS) matrix. *Staphylococcus aureus* cells within these structures are strongly protected against antimicrobial agents as well as the host defense mechanisms (del Pozo & Patel, 2007). Indeed, biofilm‐associated bacteria are up to 1,000 times more resistant to antibiotics than their planktonic counterparts (Høiby *et al*, 2010). Different biomolecules have been described to attack or prevent biofilm formation. One of the best known for *S. aureus* is the dispersin B enzyme, which is a glycosyl‐hydrolase able to break linear polymers of N‐acetyl‐D‐glucosamine present in most common *S. aureus* biofilm matrices (Kaplan, 2009). Weakening the biofilm offers the opportunity to use bacteriolytic agents, such as antibiotics or enzymes that attack the cell wall (e.g., lysostaphin, LysK or CHAPK) (Martínez de Tejada *et al*, 2012). Hence, a bacterium devoid of cell wall, such as *M. pneumoniae*, could express biofilm‐dispersing agents and bacteriolytic enzymes, making it a promising therapeutic agent against *S. aureus* biofilms.

To address this hypothesis, we first removed a panel of different pathogenic components of *M. pneumoniae* and assessed their contribution to its virulence in a mouse mastitis model (Buddle *et al*, 1984). Second, we identified the peptide signals that determine the secretion of *M. pneumoniae* proteins and designed optimal promoters to increase transcriptional and translation efficiencies (Yus *et al*, 2017). Third, we introduced a gene platform into the attenuated strain (i.e., the chassis) that codes for dispersin B (Kaplan, 2009) and lysostaphin (Bastos *et al*, 2010). Finally, the therapeutic effect of the resulting engineered strain to treat *S. aureus* biofilms was tested *in vitro*, *ex vivo*, and *in vivo* in a subcutaneous catheter mouse model (Garrido *et al*, 2014). Overall, our results showed a significant reduction of biofilm development *in vivo* in catheter‐bearing mice treated with the attenuated *M. pneumoniae* strain secreting dispersin B and lysostaphin. To our knowledge, this is the first bacterial therapy based on a genome‐reduced bacterium, and it provides an interesting framework to fight against clinically relevant biofilm‐associated bacterial infections, such as those caused by *S. aureus*.

## Results

### Rational engineering of *Mycoplasma pneumoniae* as an attenuated chassis

**Figure EV1:**
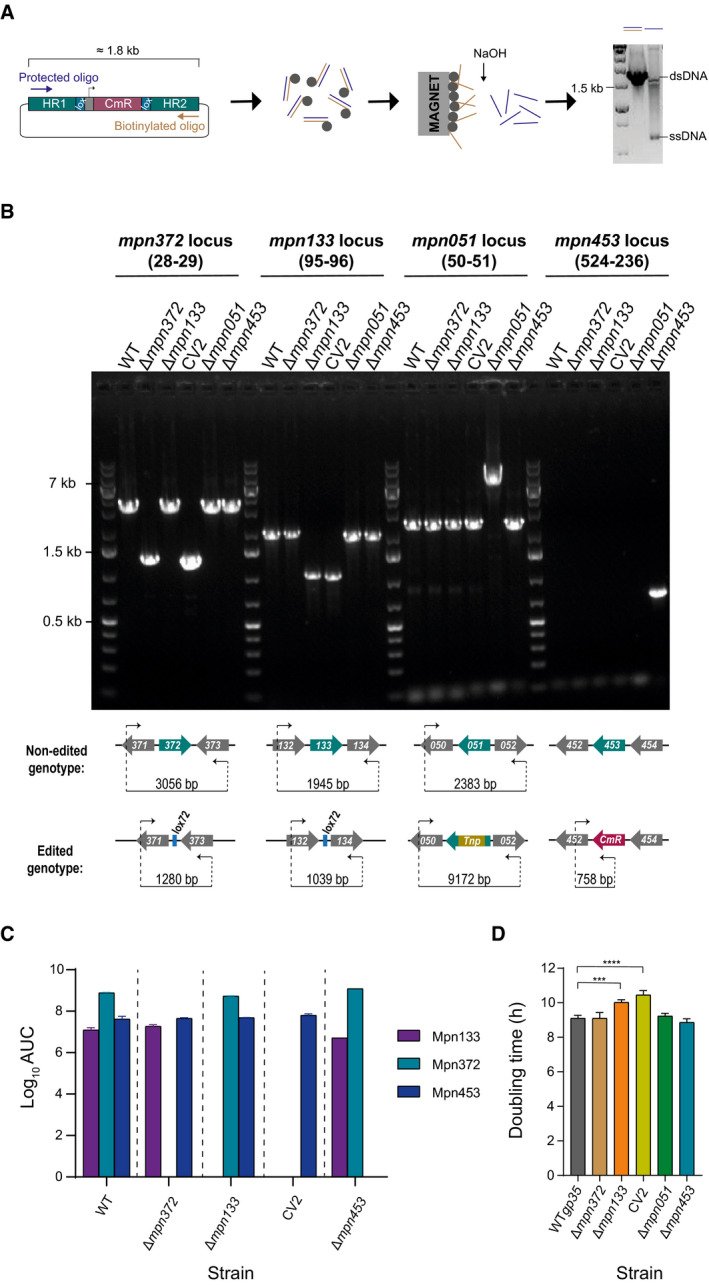
Generation and characterization of *M. pneumoniae* mutant strains Scheme depicting the protocol followed for the generation of ssDNA recombineering substrates employed to obtain the engineered strains. The illustration shows (from left to right) the amplification of dsDNA PCR products, their incubation with streptavidin‐coated magnetic beads, the NaOH‐mediated release of the strand of interest and the electrophoresis analysis of the products before and after ssDNA purification.Top, electrophoresis analysis of the PCR products obtained at each edited loci for the indicated strains. The internal code of the oligos employed for the screening is shown in brackets, and their sequences can be found in Dataset EV6. Bottom, scheme depicting the expected sizes of the PCR products if the respective locus is edited or not.Plot showing the results of the mass spectrometry analysis done for the mutant strains generated in this work. Bars represent the area under the curve (AUC) values for the three most abundant peptides of each protein in the proteome. Results are shown as the mean ± SD of two technical replicates (*n* = 2), except for ∆*mpn453* strain for which only data for one technical replicate are available. The complete data of the MS analysis can be found in the Dataset EV1. Note that the ∆*mpn051* strain was not included in this analysis, as its corresponding edited gene is disrupted by a transposon insertion, and not deleted.Plot showing the estimated doubling times of the mutant strains after 48 h of growth calculated by total protein content increase. Results are shown as the mean ± SD of three biological replicates (*n* = 3). Complete data of this analysis can be found in Dataset EV2. Results from Fisher’s PLSD test are shown for those strains that showed a doubling time statistically different from that of the parental WT*gp35* strain. ***P ≤ 0.0005; ****P ≤ 0.00005.

The pathogenesis of *M. pneumoniae* is a multilayer phenomenon, in which adhesion, nutrient depletion, and release of metabolic subproducts and toxins are suggested to be the major contributors of its cytopathic effect (He *et al*, 2016). To generate an attenuated chassis strain based on *M. pneumoniae*, we first determined the relative contribution of each of these layers to the virulence of *M. pneumoniae*. Taking advantage of the genome editing tools developed by our group based on GP35 ssDNA recombinase (Piñero Lambea *et al*, 2020), and using as substrates for recombination long stretches of ssDNA (Fig EV1A), we targeted genes involved in adhesion (*mpn453*) (Romero‐Arroyo *et al*, 1999), nutrient depletion (*mpn133*) (Somarajan *et al*, 2010), and the CARDS toxin (*mpn372*) (Kannan & Baseman, 2006). In addition, we also analyzed a previously available mutant in which the gene involved in peroxide production (*mpn051*) is disrupted by a transposon insertion (Hames *et al*, 2009). The genotype of the mutant strains was confirmed by PCR analysis at each edited locus (Fig EV1B), and gene deletions performed in each strain generated in this work were further corroborated by the absence of the corresponding protein in a mass spectroscopy (MS) analysis (Fig EV1C and Dataset EV1). Doubling times of all the strains were calculated by measuring the increase in protein content after 48 h of growth (Fig EV1D and Dataset EV2). Similar doubling rates were observed (˜9 h) for all the strains except for those carrying deletions in *mpn133* gene, in which the doubling rates were slightly but significantly increased (˜10 h), as compared to the wild‐type (WT) strain.

### Evaluation of the virulence of mycoplasma mutants in mice

**Figure 1:**
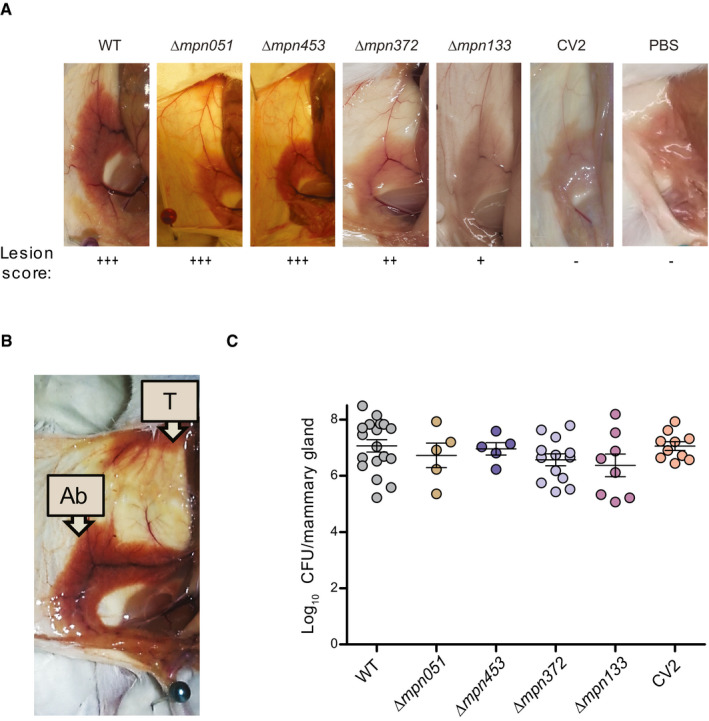
Assessment of the virulence of *M. pneumoniae* mutant strains in mouse mammary glands Representative images of abdominal mammary glands found in mice at 4 days post‐inoculation of the indicated strains. Lesion score from negative (–) to maximum (+++) is shown below each picture.Intensity and extension of the hemorrhagic lesions found in the abdominal (Ab) and thoracic (T) mammary glands of mice infected with WT bacteria.Graph showing log_10_ CFU/gland at 4 days post‐inoculation with the indicated strains (*n* ≥ 5). Each circle represents the values obtained in individual animals, whereas mean ± SD is represented with lines inside each group. No statistical differences were found between infection groups using a one‐sided ANOVA followed by the post‐hoc Fisher’s PLSD test.

**Figure EV2:**
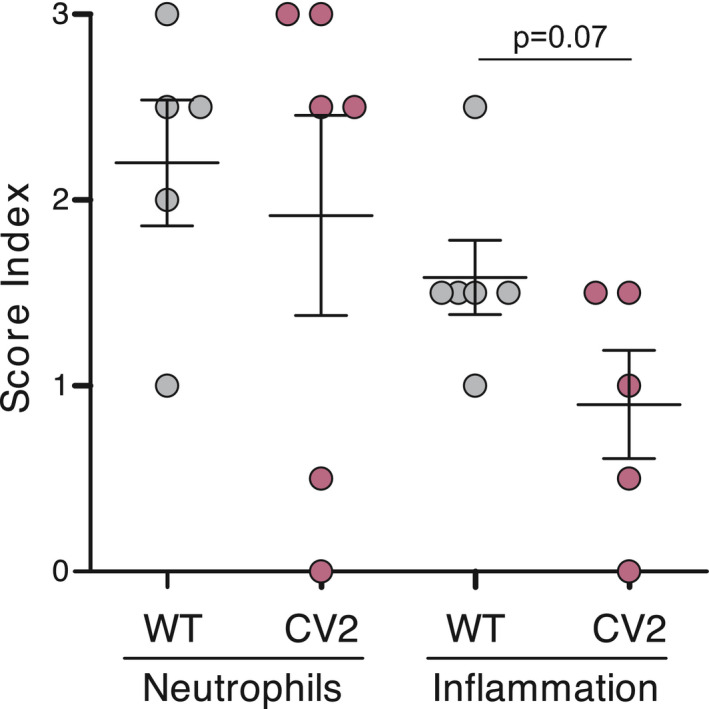
Histological score of mouse mammary glands infected with *M. pneumoniae* WT and CV2 strains Plot showing the results of the blind histopathological analysis carried out at 4 days post‐infection of the abdominal mouse mammary glands. Samples were excised and processed by hematoxylin‐eosin staining and scored (0–3) for the presence of neutrophil infiltration and interstitial inflammation. Each circle represents the scored assigned in individual samples (*n* ≥ 5), whereas mean ± SD is represented with lines inside each group. No statistical differences were found between infection groups by Fisher’s PLSD test, although a strong statistical tendency (*P* = 0.07) was found for interstitial inflammation as shown in the graph.

To assess the *in vivo* attenuation of the mutants generated, we chose a mouse model of mammary gland infection that was useful to establish different degrees of virulence of ovine respiratory mycoplasmas *Mycoplasma ovipneumoniae* and *Mycoplasma arginini* (Buddle *et al*, 1984). Four days after intramammary inoculation, WT and the ∆*mpn051*, ∆*mpn453*, ∆*mpn372*, and ∆*mpn133* mutants showed three different levels of macroscopic lesions, which were scored qualitatively from +++ to – (Fig 1A). The experimental groups receiving the WT strain, as well as those receiving ∆*mpn051* or ∆*mpn453* mutants, developed intense hemorrhagic lesions in the mammary glands and surrounding tissues (Fig 1A), affecting not only to the fourth right (R4) mammary gland but also the thoracic glands (Fig 1B). The pathogenic effect was clearly less intense and limited to the R4‐R5 mammary gland in mice inoculated with ∆*mpn372* and even less pronounced in those inoculated with ∆*mpn133* (Fig 1A). In light of these results, we decided to construct a double‐mutant ∆*mpn133*∆*mpn372* (termed CV2). The CV2 strain did not induce any mammary gland lesions (Fig 1A), and the mammary tissue looked as healthy as that treated with phosphate buffer saline (PBS). We sequenced the genome of CV2 strain (see Materials and Methods) to validate the deletions and rule out unwanted genome changes, confirming the expected genotype. A blind analysis to evaluate the microscopic lesions from tissue infected by WT or CV2 revealed that interstitial inflammation showed a tendency to be lower in CV2‐ than in WT‐treated animals (Fig EV2; *P* = 0.07).

Finally, bacterial loads were determined by colony‐forming units (CFUs) in the R4 and R5 mammary glands of CD1 mice after 4 days of infection. Similar bacterial loads were obtained from animals infected by different strains (Fig 1C). Thus, attenuation observed in CV2 strain was not associated with limited bacterial persistence. We then evaluated the diffusion of the WT or CV2 strains from the mammary gland to the lung, kidney, spleen, liver, or blood, by spreading a homogenate of these tissues on agar plates. No colonies were found, confirming that the mycoplasma cells did not diffuse away from the site of delivery.

In summary, we found that: (i) P‐30 adhesion factor (*mpn453*) and GlpD‐mediated peroxide production (*mpn051*) are not major contributor factors to *M. pneumoniae* virulence *in vivo* in mouse mammary glands; and that (ii) the cytotoxic nuclease (*mpn133*) and CARDS toxin (*mpn372*) contribute to *M. pneumoniae* virulence in this tissue. Overall, these results demonstrated differences in virulence between the *M. pneumoniae* strains and the safety of the CV2 strain, which are not due to differences in bacterial load or viability.

### Evaluation of the innate and adaptive immune response in mice induced by *Mycoplasma pneumoniae* mutant strains

Next, we analyzed the inflammatory immune response induced by the CV2 chassis, in comparison with that induced by the WT strain or PBS. To this end, the expression of a wide panel of cytokines and inflammatory modulators that participate in the defense and subsequent repair of tissues was analyzed (Fonseca‐Aten *et al*, 2005; Wang *et al*, 2014; Zhang *et al*, 2016) by real‐time quantitative reverse transcription PCR (RT–qPCR) in the mammary gland tissue.

**Figure 2:**
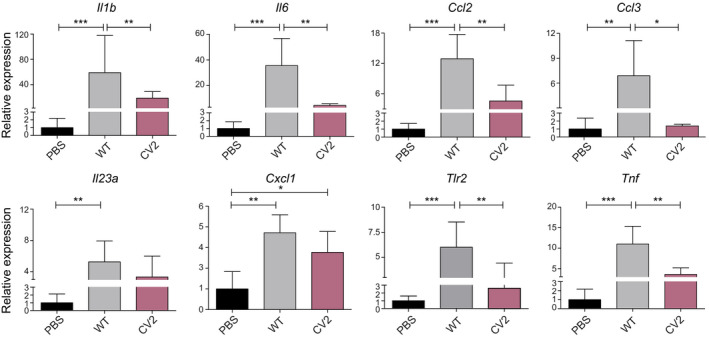
Interleukin expression profile of animals treated with WT or CV2 strains Plots showing the RT–qPCR analysis conducted to quantify relative expression of the indicated interleukins coding genes in the mammary glands. Results are expressed as the mean ± SD (*n* = 10 for PBS, *n* = 5 for WT, and *n* = 4 for CV2) of the 2^−ΔΔCt^ relative expression values of the indicated interleukins; the value from each individual animal was calculated from three technical replicates. The values obtained in the PBS group were used as control for normalization of gene expression (= 1). Statistical analysis was performed using a one‐sided ANOVA followed by the post‐hoc Fisher’s PLSD test: (*P ≤ 0.05; **P ≤ 0.01; ***P ≤ 0.005).

**Figure EV3:**
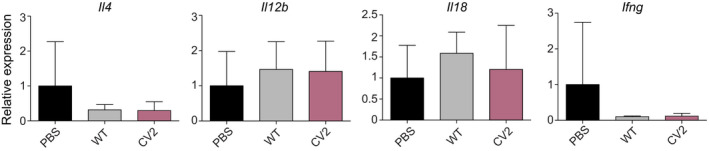
Expression profile of other interleukins in animals treated with CV2 or WT strains Plots showing the RT–qPCR analysis conducted to quantify relative expression of the indicated interleukins coding genes in the mammary glands. Results are expressed as mean ± SD (*n* = 10 for PBS, *n* = 5 for WT, and *n* = 4 for CV2) of the 2^−ΔΔCt^ relative expression values of the indicated interleukins; the value from each individual animal was calculated from three technical replicates. The values obtained in the PBS group were used as control for normalization of gene expression (= 1). Statistical analysis was performed using a one‐sided ANOVA followed by the post‐hoc Fisher’s PLSD test. No statistically significant differences were found between the different groups.

Mice receiving the WT strain showed an increased (*P* < 0.001) gene expression of all the mediators analyzed with respect to those observed in the PBS group (Fig 2), except for *Ifng*, *Il12b*, *Il4*, *and Il18* (Fig EV3). This result confirmed the pro‐inflammatory response induced by the WT strain in this experimental model. In contrast, mice administered with CV2 double mutant showed a lower gene expression (*P* < 0.05) of six of the markers analyzed (i.e., *Il1b*, *Il6*, *Ccl2*, *Ccl3*, *Tlr2*, and *Tnf*) and a statistical tendency for lower *Il23a* expression (*P* ≈ 0.05) (Fig 2), in comparison with the WT group. The only significant difference between CV2 and PBS groups was found for *Cxcl1* expression (*P* < 0.05) (Fig 2), suggesting that this response was not abrogated in the CV2 mutant.

Our results suggest that the pro‐inflammatory response triggered by *M. pneumoniae* WT could be associated with the hemorrhagic lesions phenotype observed in the mammary glands of the infected mice. Hence, the low immune response triggered by CV2 supports the non‐pathological phenotype observed in the mammary gland mouse model. This, and the fact that CV2 remained in the mammary gland for the same amount of time as the WT (Fig 1C), suggested that the CV2 strain is an excellent chassis candidate in which to introduce recombinant proteins with therapeutic purposes.

**Figure EV4:**
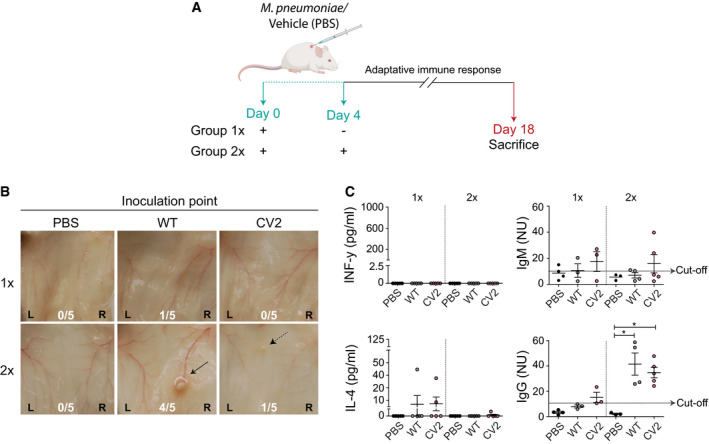
Evaluation of adaptive immune response induced by *M. pneumoniae* strains in a subcutaneous mice model Experimental design. CD1 female mice were inoculated subcutaneously with a single or repeated bacterial solution of WT or CV2 containing 1 × 10^8^ CFU/mouse at day 0 or day 0+ day 4 (referred to as group 1× and group 2×, respectively). On day 18, animals were sacrificed. The image was created with the help of BioRender.com.Macroscopic evaluation of the subcutaneous tissue of animals subjected to one (1×) or two doses (2×) of PBS, WT or CV2. The ratio of animals showing relevant findings at the inoculation point is shown within each picture (L, left; R, right).Determination of INF‐y, IL‐4, IgM, and IgG protein levels in serum samples measured by ELISA. Each circle represents the values obtained in individual animals (*n* ≥ 3), subjected to one (1×) or two doses (2×), whereas mean ± SD is represented with lines inside each group. Statistical analysis was performed using one‐way ANOVA and the Tukey post‐hoc test. *P < 0.05.

In order to evaluate whether an adaptive immune response was generated, one or two doses (on day 0 or day 0 plus day 4) of WT or CV2 strains were injected subcutaneously (Fig EV4A). The Th1 response was evaluated by measuring the levels of INF*‐y*, and Th2 response, by measuring IL‐4, IgM, and IgG levels from serum samples at the final time point (day 18). Also, a macroscopic evaluation of the inoculation point was done to evaluate for potential tissue damage.

At the macroscopic level, the single injection did not cause significant lesions in the tissue. Interestingly, after two injections, a non‐necrotic inoculation point was observed in 4 of the 5 mice inoculated with WT strain, but only in 1 of the 5 mice inoculated with CV2 strain (Fig EV4B). This result corroborates that the CV2 strain is attenuated, as previously observed in the mammary gland tissue.

Finally, we analyzed the adaptive immune response in mice serum (Fig EV4C). INF‐y was not detected in groups treated with a single or repeated doses of either strain, and IL‐4 levels were comparable between groups (Fig EV4C). Next, IgM and IgG against *M. pneumoniae* were evaluated. A low positive rate of IgM was identified, and no significant differences were observed between animals inoculated with one or two doses of WT or the CV2 strain. Interestingly, high levels of IgG were detected in mice inoculated with either strain after the second injection (Fig EV4C). All together, these results suggest that both CV2 and WT promoted an adaptive response in mice.

### Identification of secretion signals for protein expression and delivery

We decided to integrate a genetic platform in the CV2 chassis for the production and secretion of therapeutic proteins, by first identifying and then optimizing the signal sequences that promote protein secretion in this bacterium. To gain insight into the secretion system of *M. pneumoniae*, we studied its secretome by combining *in silico* and experimental approaches.

By using a dimethyl labeling approach combined with MS (Tolonen *et al*, 2011) (see Methods), we studied the secretome at two different secretion time points (24 and 72 h; two biological replicates). Essentially, we dimethyl‐labeled (using heavy, medium, or light isotopes) the proteins in the medium distinctly from the ones in the cells and determined the relative abundance of a protein in both fractions using MS (see Materials and Methods). In addition, we ran the algorithms Secretome P (Bendtsen *et al*, 2005) and Signal P 3.0 (Bendtsen *et al*, 2004) to examine the *in silico* predictions of the likelihood of each sequence to be secreted. Overall, 22 proteins were clearly enriched in the secreted fractions (summatory of secretion ratio in the four data sets > 13) (Dataset EV3). Based on the degree of enrichment in the culture supernatants, the reproducibility of this enrichment across the four datasets, and the *in silico* predictions to be secreted, we selected the N‐terminal region of 9 proteins to test their ability to promote the secretion of a heterologous protein. As a negative control, we included one cytosolic protein (Mpn322). We then fused the DNA sequences corresponding to the N‐terminal region of these 10 proteins (see Dataset EV4A for the exact sequences) to that of the catalytic domain of A1‐III alginate lyase (residues 49–402, BAB03312.1).

**Figure 3:**
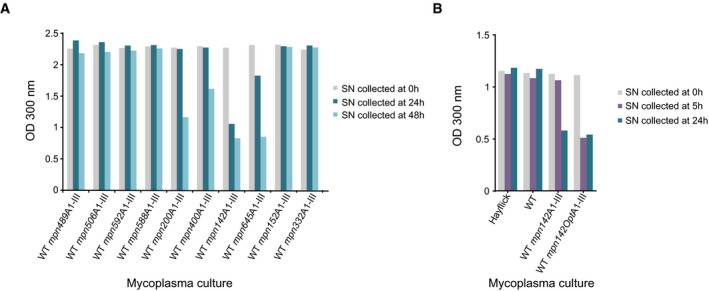
Validation of secretion signals with alginate lyase A1‐III Plot showing results of the turbidimetric assay (OD 300 nm) conducted to evaluate alginate lyase activity present in the culture supernatants (SN) of the indicated strains carrying the alginate lyase A1‐III coding sequence fused to different secretion signals. Culture supernatants (*n* = 1) were collected at 0, 24, or 48 h post‐inoculation as indicated.Comparison of the turbidimetric assay results obtained with *mpn142* and *mpn142Opt* secretion signals. Culture supernatants (*n* = 1) were collected at 0, 5, or 24 h post‐inoculation as indicated. WT strain and medium (Hayflick) were added as controls.

Cultures of the strains carrying the different secretion signals fused to A1‐III were grown in the presence of alginate substrate, and samples of the supernatants were collected at 0, 24 or 48 h after initial inoculation. Degradation of alginate substrate in these samples was determined in a turbidimetric assay (Kitamikado *et al*, 1990). We found that the construct carrying the predicted signal peptide of the *mpn142* gene showed the highest level of secreted protein, regardless of the window of time selected to collect the culture supernatant (Fig 3A). Some of the fused proteins did not show detectable activity of A1‐III in the secreted fraction, even though a very significant secretion ratio was found for the native protein (e.g., Mpn592), suggesting that the functional secretory signal of the protein might be larger than the N‐terminal region selected for the screening. To improve the levels of protein production, we designed a new *mpn142*‐based secretion signal (*mpn142Opt*) with a minimized secondary structure in the 5′ of the mRNA. To this end, the codons for the secretion signal were changed from the WT sequence following the recommendations of the company DNA 2.0. Cultures of WT and strains carrying *mpn142* or *mpn142Opt* secretion signals fused to A1‐III were then grown in the presence of alginate substrate, and samples of their supernatants were collected at 5 or 24 h after inoculation (Fig 3B). Supernatants from both strains collected at 24 h post‐inoculation showed similar levels of alginate lyase activity. In contrast, of the supernatants collected at 5 h post‐inoculation, only that of *mpn142Opt* strain showed detectable alginate lyase activity (Fig 3B), suggesting higher levels of secreted protein in this strain.

### Efficacy of *in vitro* and *ex vivo* dispersal treatments of *S. aureus* biofilm formed on plates and catheters

*Staphylococcus aureus* is a frequent colonizer of medical implants and indwelling devices, such as catheters, to which the bacteria can be easily attached and produce a biofilm matrix. Biofilm architecture and composition differ greatly between *in vivo* and *in vitro* conditions (Bjarnsholt *et al*, 2013). To implement biofilm dispersal activity in mycoplasma, we designed a genetic platform based on the *mpn142Opt‐*derived secretion signal fused to a protein with antibiofilm activity (dispersin B). This platform was first transformed into CV2 cells, generating the strain CV2‐DispB.

**Figure 4:**
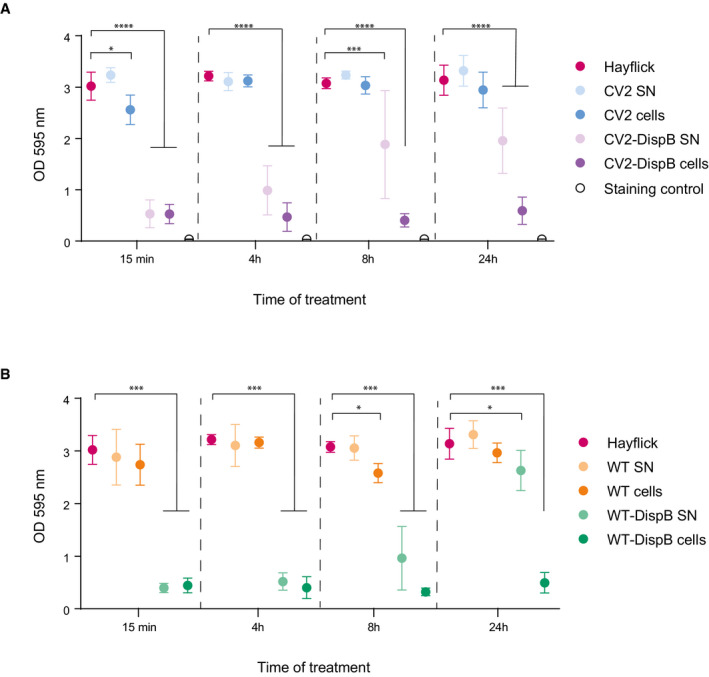
*In vitro* dispersion assay of *S. aureu*s mature biofilms formed in microplates A, BMature *S. aureus* biofilms were allowed to develop for 24 h in polystyrene plates and then treated for the indicated time intervals with cell suspensions, or culture supernatants of the CV2 or the CV2‐DispB strains (A) or the WT or the WT‐DispB strain (B). Biofilm presence was assessed by crystal violet staining and included a negative staining control (i.e., crystal violet without biofilm). The results are expressed as mean ± SD of OD 595 nm absorbance values obtained from three different biological replicates (*n* = 3) and statistically compared by one‐sided ANOVA followed by the post‐hoc Fisher’s PLSD test: *P ≤ 0.05; **P ≤ 0.005; ***P ≤ 0.0005; ****P ≤ 0.00005.

We tested the ability of CV2‐DispB to dissolve *S. aureus* 24‐h mature biofilms *in vitro* by adding CV2‐DispB supernatant (CV2‐DispB SN) or directly adding CV2‐DispB cells (CV2‐DispB cells) to polystyrene wells containing the preformed biofilm (Fig 4A). Staining with crystal violet showed that, at 15 min after treatment, both supernatant and cells‐based treatments drastically reduced the biofilm integrity (Fig 4A). Nonetheless, when the biofilm integrity was evaluated at longer exposure intervals, the effectiveness of CV2‐DispB SN treatment decreased with respect to the 15‐min time point, suggesting that the activity of secreted recombinant Dispersin B is lost over time. In contrast, treatments based on CV2‐DispB cells were highly effective throughout the experimental time, with a sustained reduction of the biofilm (Fig 4A). Similar results were obtained when the platform was introduced in the WT strain (Fig 4B).

**Figure 5:**
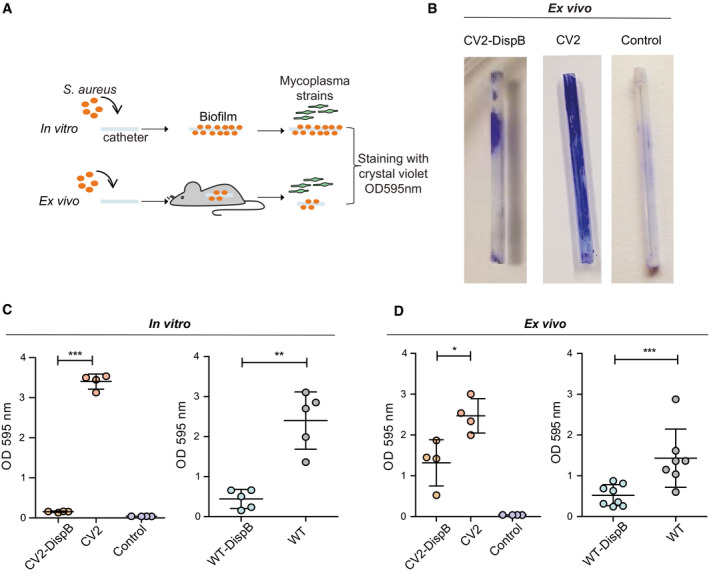
*In vitro* and *ex vivo* dispersion assays of *S. aureus* mature biofilms formed on sealed catheters ASchematic representation of the experimental procedure. Catheters pre‐colonized with *S. aureus* were allowed to form biofilms by incubation at 37°C (for *in vitro* dispersion assay) or by subcutaneous implantation in mice (for *ex vivo* dispersion assay) before being treated *in vitro* with different mycoplasma strains and estimate biofilm dispersion by crystal violet staining.BRepresentative pictures of catheters from the *ex vivo* dispersion assay with the indicated strains after crystal violet staining are shown. A staining control based on catheters in which no biofilm is formed was also included.C, DPlots showing results obtained from the *in vitro* or *ex vivo* dispersion assays with the indicated strains and from the staining control. Each circle represents the OD 595 nm values obtained for individual catheters (*n* ≥ 4), whereas mean ± SD is represented with lines inside each group. Results from one‐sided ANOVA followed by Fisher’s PLSD test are shown for treatments statistically different from those based on strains not secreting dispersin B (i.e., the WT or CV2 strains). *P ≤ 0.05; **P ≤ 0.005; ***P ≤ 0.0005.

We also tested the usefulness of our *M. pneumoniae* dispersin B platform against either *in vitro* or *in vivo* formed *S. aureus* biofilm developed on catheters (Fig 5A). The *S. aureus*‐infected catheters were surgically excised from mice and treated *ex vivo* for 4 h with CV2‐DispB cells, using the CV2 strain as a control, and biofilm dispersion was quantified by crystal violet staining. A representative picture of the catheters after the *ex vivo* treatment is shown (Fig 5B). The catheters treated with CV2‐DispB strains, but not those treated with CV2, showed a significant dispersion of biofilms formed not only *in vitro* (Fig 5C) but also formed *in vivo* and subsequently treated *ex vivo* (Fig 5D). Similar results were obtained when comparing the WT‐DispB to the WT strain (Fig 5C and D). Overall, these results highlight the capacity of the CV2‐DispB attenuated chassis to disrupt *S. aureus* biofilms, not only *in vitro* but also *ex vivo*.

### *In vivo* treatment of mice carrying catheters colonized by *S. aureus*

**Figure 6:**
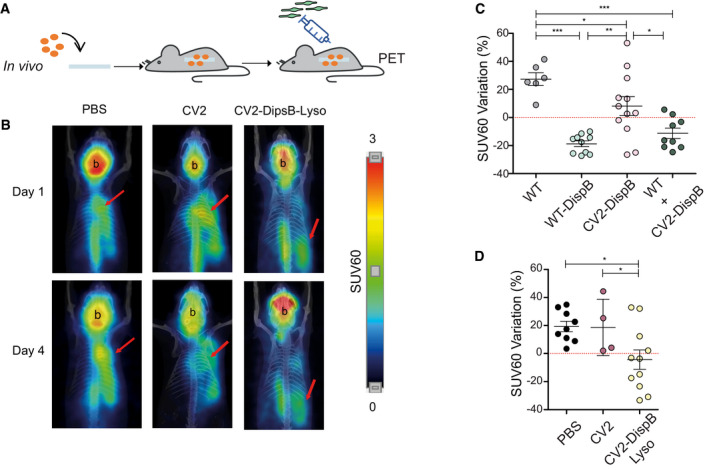
*In vivo* dispersion assay of *S. aureus* mature biofilms formed on catheters ASchematic representation of the experimental procedure. Catheters pre‐colonized with *S. aureus* were allowed to form biofilms in an *in vivo* context by subcutaneous implantation in mice. 24 h post‐implantation mice were treated with a single subcutaneous injection of different mycoplasma strains and the effectiveness of each treatment was followed by positron tomography with [^18^F]‐FDG‐MicroPET.BRepresentative images of longitudinal slices of [^18^F]‐FDG‐MicroPET uptake in mice carrying implanted catheters (red arrows) at day 1 or day 4 of the treatments. Micro‐PET images have been superimposed with CT‐3D images used as anatomical reference. Brain location is highlighted (b).C, DPlots showing the SUV60 variation (%) between day 1 and day 4 of the different treatments. Each circle represents the SUV60 variation obtained for individual animals (*n* ≥ 4), whereas mean ± SD is represented with lines inside each group. Data below the dotted lines indicate that the SUV 60 values decreased at D4 post‐treatment. Results from one‐sided ANOVA followed by Fisher’s PLSD test are shown. *P≤ 0.05; **P ≤ 0.005; ***P ≤ 0.0005.

Next, we examined whether WT‐DispB and/or CV2‐DispB cells were able to dissolve *S. aureus* biofilms *in vivo*. To this end, CD1 mice carrying subcutaneous catheters with biofilms developed *in vivo* were treated subcutaneously with a single dose of different *M. pneumoniae* strains administered subcutaneously into the surrounding area of the catheter (Fig 6A). Mice treated with CV2, WT, or PBS were used as negative controls. Each group was assayed twice in separate experiments, to ensure the reproducibility of the model and obtain statistically robust results. All animals were analyzed by positron tomography with [^18^F]‐FDG‐MicroPET, at both 1 and 4 days post‐treatment, as detailed previously (Garrido *et al*, 2014) (Fig 6B). To obtain a qualitative estimate of the bacterial abundance, immune cell infiltration, and inflammation, we determined the SUV60 signal (see Methods). The efficacy of the treatment was expressed as the percentage of variation of the SUV60 signal obtained in the catheter area from 1 to 4 days after treatment. In line with previous results (Garrido *et al*, 2014), mice treated with PBS showed an increased SUV60 signal of around 20% (Fig 6B), an indicative of the virulent progress of the *S. aureus* biofilm infection. Administration of WT or CV2 did not induce any therapeutic effect, showing 25 and 20% of SUV60 increasing values, respectively (Fig 6C and D). In contrast, all mice treated with WT‐DispB cells showed a significant (*P* < 0.001) decrease of around 20% in the PET signal (Fig 6C), a drop that according to previous studies can be associated with a decrease of around two orders of magnitude of *S. aureus* living cells (Garrido *et al*, 2014). Unexpectedly, the therapeutic effect of WT‐DispB strain could not be replicated in mice treated with the CV2‐DispB strain (Fig 6C). Since the *in vitro* and *ex vivo* treatments (above) showed that CV2‐DispB was as effective as WT‐DispB to disperse biofilms *in vitro*, we investigated whether the lesions and/or the inflammatory response evidenced by WT in the mammary gland model played a key role in the efficacy of our *M. pneumoniae* platform. For this, we included one group of CD1 mice treated with a mix (1:1) of WT and CV2‐DispB strains (Fig 6C). While these two strains individually were unable to efficiently disperse *S. aureus* biofilms, their co‐administration resulted in a treatment as efficient as that with the strain WT‐DispB (Fig 6C), indicating that the inflammatory response triggered by Mpn133 and Mpn372 proteins might have a role in clearing the infection. Altogether, these results suggested that *in situ* release of dispersin B by the CV2 strain is not sufficient in itself to control an *S. aureus* catheter infection *in vivo*.

### Implementation of antimicrobial activity on the gene platform against *S. aureus* biofilms

To avoid any requirement for an inflammatory response in order to remove an *S. aureus* infection, we tested if expressing a bacteriolytic agent in combination with dispersin B in the CV2 strain could remove *S. aureus* infection, without the adverse effects. We selected the glycylglycine endopeptidase lysostaphin, which cleaves the pentaglycine crossbridge of the staphylococcal cell wall, killing *S. aureus* and affecting biofilms *in vitro* and *in vivo* (Wu *et al*, 2003; Kokai‐Kun *et al*, 2009).

**Figure EV5:**
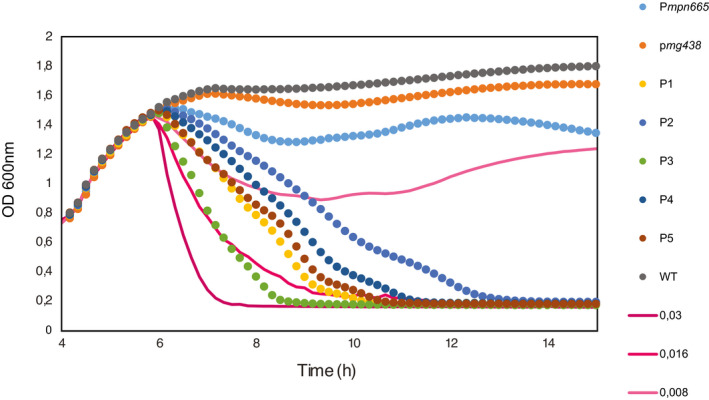
Impact in *S. aureus* growth curves of lysostaphin production by *M. pneumoniae* driven by different promoter sequences Plot showing a growth curve of *S. aureus*. After 6 h of growth, 20 µl of different treatments was added. Dotted lines represent treatments based on culture supernatants (*n* = 1) of different *M. pneumoniae* strains carrying the *mpn142Opt*‐Lysostaphin construct under control of the indicated promoter sequences. A treatment based on the supernatant of a culture of WT strain was added as control. Continuous lines represent treatments based on recombinant lysostaphin protein at the indicated concentrations in µg/µl.

First, we characterized the expression of lysostaphin by *M. pneumoniae*. For this, we obtained different strains by introducing the lysostaphin coding gene under naturally existing (P*mpn665* and P*mg438*) promoters, or a set of different synthetic (P1 to P5) promoters (Dataset EV4B), whose design was based on the rules governing efficient transcription and translation in this bacterium (Yus *et al*, 2017). We then checked the bacteriolytic activity of different strains on *S. aureus* growth curves. Strains with endogenous promoters showed minor effects on *S. aureus* growth. In contrast, we identified the P3 synthetic promoter as the one producing the highest lysostaphin activity, as inferred from the drastic effect of the strain carrying this promoter in *S. aureus* growth (Fig EV5).

We next transformed CV2‐DispB cells with a transposon vector harboring the P3_*mpn142Opt*_lysostaphin construct, to generate the CV2‐DispB‐Lys strain. The ability of this strain to disrupt the progression of *S. aureus* biofilms *in vivo* was assessed as before by [^18^F]‐FDG‐MicroPET images in CD1 mice carrying colonized catheters. Indeed, mice treated with CV2‐DispB‐Lys cells showed impaired biofilm progression with respect to those receiving CV2‐DispB or CV2 (Fig 6C and D). These results confirmed that the attenuated CV2 strain could represent an attractive chassis vector against *S. aureus* biofilms if the expression of a biofilm dispersal agent is combined with an antimicrobial peptide.

## Discussion

Biofilm‐associated infections account for up to 80% of hospital‐acquired infections, and *S. aureus* is one of the leading species in this regard. These infections are usually associated with different medical implants, such as catheters, prosthetic joints, and cardiac pacemakers. As biofilms form structures that are highly resistant to antibiotics and environmental stresses, there is an urgent need to develop novel therapies against biofilm‐associated infections.

The use of reprogrammed bacteria to treat different diseases is gaining attention as a promising therapeutic approach. In fact, several bacterial therapies have been developed to target different infectious agents, such as *Pseudomonas aeruginosa* (Saeidi *et al*, 2011; Gupta *et al*, 2013), *Vibrio cholerae* (Duan & March, 2010; Jayaraman *et al*, 2017), *Enterococcus faecalis* (Borrero *et al*, 2015), *Salmonella enterica* (Palmer *et al*, 2018), and *Mycobacterium smegmatis* (Atanaskovic *et al*, 2014). Nevertheless, most of these approaches have not been tested in *in vivo* models and are intended to destroy planktonic bacteria, which are not as clinically relevant as biofilm‐associated bacteria. Only an engineered *E. coli* Nissle strain has been specifically designed to destroy *P. aeruginosa* biofilms, producing remarkable results in the gastrointestinal tract of a murine model (Hwang *et al*, 2017). However, the versatility of this *E. coli* Nissle platform as a therapeutic agent against pathogens is limited, since the presence of cell wall on this bacterium imposes a restriction to its ability to secrete molecules or to be used in combination with antibiotics or protein products targeting this structure. In contrast, given the absence of a cell wall, our platform based on a *M. pneumoniae‐*derived CV2 strain would enable the secretion of a wide variety of molecules targeting this structure. Moreover, it can be co‐administered with antibiotics that are directed against cell wall formation, which might boost the efficacy of the treatments and allow antimicrobial effect of otherwise discarded antibiotics against biofilm‐associated infections to be rescued.

Given the pathogenic character of *M. pneumoniae*, the first step we took was to generate an attenuated version, called CV2. Of note, some genes acknowledged as drivers of *M. pneumoniae* pathogenesis in the lung are kept in our attenuated CV2 strain. This falls in line with the increasing evidence, suggesting that pathogenicity is highly context‐dependent (Casadevall & Pirofski, 2001). For instance, adhesion to the host cell has been demonstrated as an unavoidable requisite for *M. pneumoniae* cells to start an infection on lung cell lines. However, the deletion of *mpn453* gene did not produce a significant reduction of the hemorrhagic phenotype in our experimental mouse mammary gland model. Moreover, the number of CFU recovered from the infected tissue at 4 days post‐infection was similar between this non‐adherent strain and its adherent counterparts (Fig 1C). These results suggest that P30‐mediated adhesion is dispensable in our experimental model. Likewise, the deletion of the *mpn051* gene that generates H_2_O_2_ in the presence of glycerol did not result in an attenuated phenotype in our screening. Glycerol metabolism might be especially active on the respiratory tract, where phosphatidylcholine is abundant as a major component of the pulmonary surfactant (Agassandian & Mallampalli, 2013). In contrast, glycerol does not seem to be the major carbon source available in subcutaneous settings, as indicated by the ability of the strain lacking *mpn051* to persist in this environment. Hence, we decided to use the CV2 strain (i.e., a *M. pneumoniae* strain lacking the *mpn372* and *mpn133* genes) as an attenuated version suitable to be complemented with a genetic platform against *S. aureus* biofilms. It should be noted that in spite of the limited inflammatory response triggered by CV2 cells (Fig 2), our results indicate that repeated injections of CV2 cells generate an IgG response (Fig EV4), which could potentially affect the efficacy of long‐term treatments or those involving repeated use. However, this IgG response might not necessarily limit the efficacy of CV2‐based treatments in a subcutaneous setting, as mycoplasma‐associated infections are characterized by the subversion of host immune responses (Qin *et al*, 2019). In any case, this is an interesting result, as so far most of the bacterial therapies available are targeted to the gastrointestinal tract, an environment in which the immune system has evolved to establish tolerance toward an enormous and constantly changing amount of microorganisms (Zheng *et al*, 2020). It is unclear whether such an immune tolerance mechanism is also present in the respiratory tract, which is the natural niche of *M. pneumoniae*, but an increasing amount of data suggesting the existence of a resident microbiome also in this tissue might point to this direction (Dickson *et al*, 2016).

In a second step for adapting CV2 as a chassis capable of *in situ* release of therapeutic compounds, we identified the secretory signals of this bacterium. Three main pathways for secretion exist in bacteria: the Sec pathway associated with a signal peptidase (Beckwith, 2013), the twin‐arginine translocation (Tat) pathway (Patel *et al*, 2014), and unconventional secretion (Bendtsen *et al*, 2005). *Mycoplasma pneumoniae* does not have the Tat pathway or its associated machinery (to the best of our knowledge), but it contains the genes required for an active Sec pathway (Dandekar *et al*, 2000). Although no signal peptidase I can be identified at the sequence level, there is experimental evidence, suggesting that such an activity is present in this bacterium (Catrein *et al*, 2005). Additionally, there are experiments suggesting unconventional secretion (Dallo *et al*, 2002; Balasubramanian *et al*, 2008). In this work, we identified that the signal peptide of *mpn142* gene can promote the secretion of heterologous proteins in the *M. pneumoniae* chassis. Signal P 3.0 server identifies a cleavage site between the residues 25–26, corresponding to the sequence SLA‐NT. This sequence has properties similar to those found in the Sec‐signal peptidase pathway, corroborating that there is a signal peptidase I‐like activity in *M. pneumoniae* (Catrein *et al*, 2005). Whereas secretion signal based on *mpn142* showed the highest activity of alginate lyase in the culture supernatant specially at short time points (Fig 3), other signals (such as *mpn645*, *mpn400*, and *mpn200*) seemed to be also functional and might be of interest when designing more complex genetic platforms involving the secretion of several proteins.

The *in vitro* experiments with the CV2‐DispB strain highlight one the main advantages of bacterial therapies, which is the possibility of a continuous and localized production of therapeutic doses of a given protein. Specifically, whereas treatments based on supernatants were able to affect biofilms only over a short term, treatments with cells led to long‐term disruption of biofilms (Fig 4). The fact that the supernatant effect over *S. aureus* biofilms was not sustained over time suggests that dispersin B activity has a limited half‐life. Supernatants contain a high but non‐renewable amount of dispersin B, a situation that might be similar to the one encountered when recombinant proteins are administered as a treatment. In contrast, cells represent a source for the continuous supply of dispersin B. Given the apparent short half‐life of this enzyme, bacterial‐based delivery of dispersin B provides an advantage over the use of recombinant protein, not only in terms of production cost but also (and more importantly) in terms of treatment efficacy.

Finally, in contrast to the *in vitro* and *ex vivo* experiments done with catheters, in which WT‐DispB and CV2‐DispB behaved similarly (Fig 5), the *in vivo* experiments revealed that WT‐DispB cells, but not CV2‐DispB cells, effectively dissolved catheter‐associated biofilms. However, the co‐administration of two strains that separately were unable to dissolve *S. aureus* biofilms (i.e., WT and CV2‐DispB strain) produced a significant reduction on the PET signal (Fig 6). Collectively, these results indicated that dispersin B release was necessary but not sufficient to mediate biofilm dispersion and that the presence of WT cells is required for successful biofilm reduction. This requirement for the WT strain might be due to the presence of the *mpn133* gene, which is deleted in the CV2 strain. This gene encodes for a nuclease, and DNA is known to be an important component of biofilms. However, whereas different nucleases have proven to be effective biofilm dispersal agents in *in vitro* conditions, their effect on biofilms developed *in vivo* seems to be limited (Kaplan, 2009). Additionally, *M. pneumoniae* encodes for a second nuclease (Mpn491) that has been reported to facilitate evasion from neutrophil extracellular traps (NETs) (Yamamoto *et al*, 2017). Thus, it is unlikely that the deletion of *mpn133* gene is responsible for the impaired ability of CV2 strain to dissolve biofilms in *in vivo* conditions. Alternatively, WT cells, but not CV2 cells, seem to trigger an inflammatory response (Fig 2). This is of special interest, considering the numerous immune evasion strategies that *S. aureus* has acquired (de Jong *et al*, 2019). In this scenario, CV2‐DispB cells would be able to dissolve the biofilm, but *S. aureus* cells detached from this structure would be still able to recolonize the catheter. In the case of the WT‐DispB, the inflammatory response triggered by the WT strain would be somehow responsible for eliminating the *S. aureus* cells detached from the catheter.

We were able to rescue the CV2‐DispB strain as an effective therapeutic agent against biofilms by implementing an antimicrobial activity into its gene platform. We chose Lysostaphin as an antimicrobial activity, as an example of a bacteriocin that shows a high degree of specificity against *S. aureus* cells that is therefore unlikely to interfere with other beneficial bacteria. Our results suggest that in those mice treated with the CV2‐DispB‐Lys strain, the cells detached from the biofilm by means of dispersin B activity are killed by lysostaphin. Therefore, the inclusion of the antimicrobial activity seems to impede recolonization of the catheter and to replace the role of the inflammatory response caused by WT‐DispB strain. Although the inclusion of lysostaphin clearly improved the outcome of the treatment compared to CV2‐DispB cells, the efficacy of the CV2‐DispB‐Lys strain in reducing biofilm‐associated catheters was less consistent than that of WT‐DispB cells. Several reasons could explain the inconsistency in the outcome of treatments based on CV2‐DispB‐Lys cells. One is inherent to the model of the subcutaneous catheters, as it depends on the behavior of the particular mouse; here, the catheter might move away from the place in which mycoplasma was administered, hindering the therapeutic effect. Indeed, the results obtained with the WT‐DispB strain are more consistent between different animals, which might suggest that inflammation created by WT strain limits the movement of the catheter. Of note, this subcutaneous setting is not the natural niche of *M. pneumoniae*, which probably limits its capacity to spread within the tissue and to compensate for the possible movement of the catheter. Therapies based on systemic administration of recombinant proteins might not face this limitation but are certainly more prone to adverse effects. An alternative explanation for this inconsistency is the possible appearance of resistance to lysostaphin, a phenomenon that is quite common in *S. aureus* (Gründling *et al*, 2006). Hence, a possible way to improve a therapy based on the CV2 strain would to combine dispersin and lysostaphin with other antibacterial peptides (e.g., CHAPK, LysK), thereby overcoming any possible appearance of resistance by overwhelming the adaptation capacity of pathogenic bacteria. This is probably the biggest advantage of bacterial therapies as compared to phage therapies, which have already shown promising results against different bacteria (Lu & Collins, 2007; Khalifa *et al*, 2015), as the amount of heterologous proteins that can be expressed by a bacterial vector is virtually unlimited.

Finally, as for any other bacterial vector, biocontainment strategies should be taken into consideration before moving into the clinics. As a first step, it is important to note the subcutaneous setting employed in this study it is not the natural habitat of *M. pneumoniae*, which should presumably limit its persistence for extended periods of time. In any case, several strategies could be taken to control its growth on demand. For instance, many antibiotics are capable of limiting the growth of *M. pneumoniae*, including macrolides, tetracyclines, and fluoroquinolones (Pereyre *et al*, 2016). Alternatively, genetically encoded biocontainment strategies might be included, given the availability of inducible promoters for mycoplasma (Breton *et al*, 2010) that can control the expression of essential components of cell machinery. Even sophisticated kill switches already developed for other bacteria (Chan *et al*, 2016; Stirling *et al*, 2017) might be eventually adapted to *M. pneumoniae*.

In summary, this work provides a framework for the development of bacterial therapies based on less well‐characterized yet highly interesting microorganisms, such as for instance genome‐reduced bacteria. We foresee future applications of this technology for the treatment of biofilm‐associated respiratory infections, given the natural ability of *M. pneumoniae* to colonize the human respiratory tract.

## Materials and Methods

### Reagents and Tools table

**Table:** 

Reagent/Resource	Reference or Source	Identifier or Catalog No.
**Experimental Models**		
*Mycoplasma pneumoniae* strain WT	Richard Herrmann lab	ATTC 29342, subtype 1 (M129‐B7, broth passage no. 35)
Other *Mycoplasma* strains	This study	DataSet EV5
*Staphylococcus aureus*	Valle *et al* (2003)	Strain 15981
*Escherichia coli*	New England Biolabs	C2987H
Mice	Charles River Laboratories	CD1
**Recombinant DNA**		
Dispersin B (*Aggregatibacter actinomycetemcomitans*)	Kaplan (2009)	Synthetic gene optimized for *M. pneumoniae*. Synthesized by IDT
Lysostaphin (*Staphylococcus simulans*)	Bastos *et al* (2010)	Synthetic gene optimized for *M. pneumoniae*. Synthesized by IDT
Poly‐M‐alginate lyase A1‐III (*Sphingomonas* spp.)	GenBank: BAB03312.1	Synthetic gene optimized for *M. pneumoniae*. Synthesized by IDT
**Oligonucleotides and other sequence‐based reagents**		
Description of vectors designed to obtain the engineered strains	This study	Dataset EV6A. Synthesized by IDT
Primers used in the cloning to obtain the vectors described in section	This study	Dataset EV6B. Synthesized by IDT
Primers used for detecting cytokines by qPCR	This study	Dataset EV7. Synthesized by Sigma
**Chemicals, Enzymes, and other reagents**		
Hayflick modified medium	Yus *et al* (2009)	
Minimal medium	Yus *et al* (2009)	
(2‐hydroxy)propyl‐β‐cyclodextrin (Hyprop)	Sigma	H107
LB	BD Difco	11778902
LB‐Agar	BD Difco	11758902
TSA	CondaLab, Spain	1068
TSB	CondaLab, Spain	1224.00
D‐Glucose monohydrate	VWR ProLabo	14431‐43‐7
BactoAgar	BD Difco	214010
Ampicillin sodium salt	VWR	0339‐EU
Chloramphenicol	Sigma	C0378‐5G
Gentamicin	Gibco	15710049
Tetracycline	Sigma	87128
Puromycin	Gibco™	A1113803
[^18^F]‐FDG	Garrido *et al* (2014)	Prepared from commercially available automatic module (Synthera, IBA)
Acetone	Sigma	179124
Agarose	VWR	9012‐36‐6
Crystal violet	PanReac AppliChem	251762.1606
DD445	AB Laboratorios de Biotecnología	DD 445‐plus
DTT	Merck	10197777001
EDTA (disodium salt dehydrate)	Amresco	105
Formaldehyde solution 10% stabilized with methanol	PanReac Applichem	143091.121
Complete protease inhibitor cocktail	Roche	11836153001
Glycogen	Roche	10901393001
Iodoacetamide	Sigma	I1149
Isoflurane IsoVet®	Braun	468660
Isopropanol	Merck	1096342511
Ketamine (imagene 100 mg/ml)	Merial	
Petrolatum	Sigma	16415
StrataClean resin	Agilent Technologies	400714
Phusion DNA polymerase	Thermo Fisher Scientific	F530S
DpnI	New England Biolabs	R0176S
Gibson Assembly mix	Gibson *et al* (2009)	In‐house prepared mix
Tissue Glue	3M Vetbond^TM^	1469C
Trichloroacetic acid	Sigma	T9159
Tris Ultrapure	VWR‐Amresco	0497
Trypsin	Promega	V5113
Urea	Sigma	U5378
Xylacine (Rompum 20 mg/ml)	Bayer Health Care	
**Software**		
Graph Pad software Version 8 for windows	https://www.graphpad.com	GraphPad Prism, San Diego California USA
PMOD software Version 3.2	https://www.pmod.com	PMOD Technologies Ltd., Adliswil, Switzerland
SeqPurge tool (v0.1‐478‐g3c8651b)	Sturm *et al* (2016)	
SPAdes genome assembler v3.14.1	Bankevich *et al* (2012)	
QUAST v5.0.2 (Quality Assessment Tool for Genome Assemblies)	Gurevich *et al* (2013)	
NucDiff v2.0.3	Khelik *et al* (2017)	
**Other**		
BCA assay	Pierce	23225
Bioruptor	Diagenode	
Catheters Vialon® 18G 1.3⋅30 mm	Becton‐Dickinson Insyte^TM^	381244
Corning® cell culture flask	Merck	CLS430720
ChemiDoc	Bio‐Rad	
NanoDrop	Thermo Scientific	
Dynabeads^TM^ MyOne^TM^ Streptavidin C1	Invitrogen	65001
Electroporation system, Gene Pulser X cell	Bio‐Rad	1652662
Electroporation cuvettes 0.1 cm	Bio‐Rad	1652089
ELISA plates reader‐ IdAB	Labsystem	Multiskan Ex
Filtropur S 0,20 µm	Sarstedt	83.1826.001
Heavy dimethyl labeling mixture ((13CD2O, NaBD3CN)	Isotec	596388, 190020
Medium dimethyl labeling mixture (CD2O, NaBH3CN, (NaBD3CN)	Isotec, Fluka	492620 (Isotec), 71435 (Fluka), 190020 (Isotec)
Light dimethyl labeling mixture (CH2O, NaBH3CN	Sigma, Fluka	252549 (Sigma), 71435 (Fluka)
LTQ‐Orbitrap Velos mass spectrometer	Thermo Fisher	
Multidish plate (12 wells)	BD	351143
Multidish plate (96 wells)	Thermo Scientific	130188
NanoLC separation	Proxeon	
NanoSpray	Thermo Fisher	
PrimeScript^TM^ Reverse Transcriptase Kit (Perfect Real Time)	Takara	RR037A
Power Sybr® Green	Applied Biosystems	4367659
QIAquick PCR Purification Kit	Qiagen	28104
QIAquick Gel Extraction Kit	Qiagen	28704
QIAprep Miniprep Kit	Qiagen	27106
LightCycle 480 qPCR machine	Roche	
AriaMx Real‐Time PCR System	Agilent Technologies	
Reverse phase column 75 μm × 250 mm	Nikkyo Technos Co., Ltd	
RNeasy® Mini Kit	Qiagen	74104
Small animal Pet scanner	Philips Mosaic	Cleveland, OH, USA
Smart Spec^TM^ Plus Spectrophotometer	Bio‐Rad	4006221
Stomacher80 sterile bags	Seward Medical	BA6040
TB® Green Premix Ex Taq TM II (Tli RNase H Plus)	Takara	RR820A
Infinite M200 Plate reader	Tecan	
T‐10 basic Ultra Turrax®	Ika	0003737000
Natural Alginate from brown seaweed	Sigma	W201502
Mi‐Seq sequencing platform	Illumina	
NEBNext® DNA Library Prep Reagent Set	Illumina	E7370L
AgenCourt AMPure XP beads	Beckman Coulter	A63882
NEBNext® Multiplex Oligos	New England Biolabs	
KAPA Library Quantification Kit	Kapa Biosystems	KK4835
Mouse INF‐y ELISA	Biolegend	430804
Mouse IL‐4 ELISA	Biolegend	431104
Mouse *M. pneumoniae* IgM ELISA	NovateinBio	BG‐MUS11514
Mouse *M. pneumoniae* IgG ELISA	NovateinBio	BG‐MUS11512

### Methods and Protocols

#### Bacterial strains and culture conditions

All the strains used in this work are summarized in Dataset EV5. The *Mycoplasma pneumoniae* wild‐type (WT) strain M129‐B7 (ATTC 29342, subtype 1, broth passage no. 35) and all its derivatives generated in this work were grown at 37°C under 5% CO_2_ in tissue culture flasks (Corning) with Hayflick modified medium, as described elsewhere (Buddle *et al*, 1984). Hayflick broth was supplemented with tetracycline (2 µg/ml), puromycin (3 µg/ml), gentamicin (100 µg/ml), or chloramphenicol (20 µg/ml) for selection of cells as needed. When growth on a plate was required, Hayflick broth was supplemented with 0.8% bacto agar.

The *S. aureus* strain 15981 (Valle *et al*, 2003; Garrido *et al*, 2014) was cultured (37°C, 18 h) in tryptone soy agar (TSA) or tryptone soy broth (TSB) supplemented with glucose (0.25%, wt/vol) (TSA‐glc and TSB‐glc, respectively).

For cloning purposes, *E. coli* NEB^®^ 5‐alpha High Efficiency strain was grown at 37°C in LB broth or on LB agar plates supplemented with ampicillin (100 μg/ml).

#### Plasmids

All the plasmids generated in this work were assembled following the Gibson method (Gibson *et al*, 2009). When required, IDT Incorporation performed gene synthesis. Oligonucleotides were synthesized by Sigma‐Aldrich. Gene amplifications were carried out with Phusion DNA polymerase. A detailed list of the primers employed to generate the ssDNA recombineering substrates and the plasmids generated in this work (including the sequence of the different modules included on them) is available on Dataset EV6. In addition, the complete sequences of the most relevant plasmids are available in European Nucleotide Archive. The correct assembly of all the plasmids was verified by Sanger sequencing (GATC biotech).

#### Generation of Mycoplasma pneumoniae mutant strains

Mutants were generated using genome editing tools adapted to *mycoplasma* based on ssDNA recombinase GP35 (Piñero Lambea *et al*, 2020). As substrate for recombination, we used long stretches of ssDNA, produced as previously described (Burgos *et al*, 2020). Briefly, a set of plasmids termed pUC57∆mpn453, pUC57∆mpn133, and pUC57∆mpn372 were generated, in which a lox71‐cat‐lox66 cassette is enclosed by the regions flanking each target gene. To generate ssDNA recombineering substrates, the cassette of interest of each plasmid was amplified with oligos containing biotin or phosphorothioate modifications attached to the 5′ ends. After a clean‐up protocol, 20 µg of the corresponding PCR products was incubated with Dynabeads at RT for 2 h before capturing and resuspending the Dynabeads in 50 μl melting buffer (125 mM NaOH) to force the release of the DNA strand amplified with phosphorothioate‐modified primer. Next, the beads containing the strand amplified with biotin‐modified primer, were pulled down and the supernatant containing the DNA strand of interest was collected and diluted in 500 μl of neutralization buffer (60 mM NaAc in TE buffer). A second round of elution was performed and recovered to the same neutralization solution. The ssDNA was precipitated by adding 60 μg of glycogen and 1 volume of isopropanol. After 30 min of incubation at RT, ssDNA was recovered by centrifugation (18,000 *g*, 45 min at 4°C), and the pellet was washed twice with chilled 70% ethanol. The pellet was then air dried and resuspended in electroporation buffer (8 mM HEPES, 272 mM sucrose, pH 7.4). Finally, 3 µg of the corresponding ssDNA substrates was electroporated into M129‐GP35 strain (Piñero Lambea *et al*, 2020) following the transformation procedure previously described for *M. pneumoniae* (Montero‐Blay *et al*, 2019). After the pulse, cells were grown for 24 h in T75 flasks containing 25 ml of plain Hayflick medium to allow GP35‐mediated recombination. Cells were recovered and spread on Hayflick‐agar plates supplemented with 20 μg/ml chloramphenicol. The chloramphenicol resistance gene included into the engineered strains was then excised from the chromosome using the Cre/lox system. To this end, the strains were transformed with an in‐house suicide plasmid termed pGentaCre carrying the Cre coding sequence and a gentamicin resistance gene. One tenth of the transformation was inoculated into a T75 flask containing 25 ml of Hayflick supplemented with 100 μg/ml gentamicin. Cells were incubated for 5 days, to allow not only selection of transformed cells but also Cre‐mediated excision of Cm resistance cassette. After this period of time, surviving cells were scraped from the flask and seeded on Hayflick‐agar plates. Excision of cat selectable marker was confirmed by PCR. Quick extractions of gDNA were carried out by lysing 100 μl of cell suspensions (100°C 10 min) and subsequent mixing with 30 μl of Strataclean resin. After 10 min of incubation, the resin was pulled down by centrifugation (13,000 *g*, 1min) and 2 μl was used as template for the indicated PCRs (Fig EV1B).

#### DNA sequencing and genome assembly

We performed sequencing on Illumina Mi‐Seq platform at a high coverage (˜1,000×) of the genome of CV2 strain. Libraries were prepared using the NEBNext® DNA Library Prep Reagent Set for Illumina® kit according to the manufacturer's protocol. Briefly, 500 ng of DNA was fragmented to approximately 600 bp and subjected to end repair, addition of “A” bases to 3′ ends, ligation of NEBNext hairpin adapter and USER excision. All purification steps were performed using AgenCourt AMPure XP beads. Library size selection was done with 2% low‐range agarose gels. Fragments with average insert size of 660 bp were cut from the gel, and DNA was extracted using QIAquick Gel extraction kit and eluted in 15 µl EB. The adapter‐ligated size‐selected DNA was used for final library amplification by PCR using NEBNext® Multiplex Oligos for Illumina. Final libraries were analyzed using Agilent DNA 1000 chip to estimate the quantity and check size distribution and were then quantified by qPCR using the KAPA Library Quantification Kit. Libraries were loaded at a concentration of 15 pM onto a flowcell together with other samples at equal concentration (half a run) and were sequenced 2 × 300 on Illumina’s Miseq.

For genome assembly, reads were first trimmed using SeqPurge v0.1‐478‐g3c8651b with a minimum read length of 80 and default parameters for base calling quality threshold. Genome assembly was performed using SPAdes genome assembler v3.14.1 with trimmed reads as input, with default parameters. Quality of the assembly was assessed using QUAST v5.0.2 (Quality Assessment Tool for Genome Assemblies) against the NCBI reference genome or against the predicted CV2 genome. All statistics are based on contigs of size ≥ 500 bp.

Whole‐genome sequences were aligned using NucDiff v2.0.3, a tool that allows comparison of closely related sequences, and rigorous analysis of local differences and structural rearrangements.

#### Mice

Four‐week‐old CD1 male and female mice were purchased at Charles River Laboratories (France) and housed in appropriate cages with water and food *ad libitum*, under the pathogen‐free conditions at the Institute of Agrobiotechnology (authorization code ES/31‐2016‐000002‐CR‐SU‐US). For mammary gland infections, pregnancies were synchronized by dark–light circadian cycles, males were housed with the females for 3 days, and the offspring were removed at day 10 of lactation. For catheter experiments, mice were used after 2 weeks of housing. Animal handling and procedures were performed following the FELASA and ARRIVE guidelines (Kilkenny *et al*, 2010), in accordance with the current European (Directive 86/609/EEC) and National (Real Decreto 53/2013) legislations, and with the approval of the competent authority of Navarra Government (Resoluciones 281/2020 and 282/2020).

#### Assessment of chassis virulence in mice

To assess the virulence of the different mycoplasma chassis generated, we used a mouse model of mammary gland infection (Buddle *et al*, 1984) by adapting the protocol previously described (Brouillette *et al*, 2004). CD1 mice on day 10 of lactation (40 grams of body weight) were anesthetized with ketamine (100 mg/kg) and xylazine (10 mg/kg) and then intramammary infected in the R4 (right) abdominal mamma with the CV2 or WT strains. The intramammary inoculations were performed with 0.1 ml of a bacterial suspension containing ≈1 × 10^8^ CFU/mouse, through the galactophore channel of the R4 nipple, with the help of a 33‐gauge needle blunt end in a Hamilton syringe. At 4 days post‐inoculation (PI), mice were euthanized by cervical dislocation and the mammary glands inspected for intensity and extension of congestion, edema, and hemorrhagic lesions. This anatomopathological evaluation ranked lesions from negative (healthy tissues) to +++ (intense hemorrhage). Thereafter, the R4‐R5 mammary glands were aseptically removed, individually weighted, and handed out in: (i) Hayflick for bacterial counts, (ii) 10% formalin for histopathological studies by hematoxylin‐eosin, and (iii) liquid N_2_ for freezing and subsequent storage at −80°C for IL expression analysis. Bacterial counts were determined after homogenization of the tissue in sterile bags (Stomacher80), and seeding tenfold serial dilutions (100 µl by triplicate) on Hayflick plates. After 2 weeks of incubation, the number of CFU/gland was determined and log_10_ transformed and the mean ± SD of log CFU/gland were calculated for statistical purposes. Histopathological changes in the glandular tissues were evaluated in blind analysis and classified according to the integrity of the secretory acini and the excretory epithelia, presence of polymorphonuclear (neutrophils), and inflammatory cells.

#### RNA extraction and real‐time quantitative PCR analyses (RT–qPCR) of inflammatory genes

The frozen portion of the mammary gland was homogenized using Ultra‐Turrax and total RNA was isolated using RNeasy Mini Kit, following the manufacturer's instructions. RNA concentration was measured spectrophotometrically using a Nanodrop One, and RNA integrity was confirmed by 1% agarose gel electrophoresis. RNA samples with an absorbance at 260:280 nm ratios of 1.8–2.1 were used for RT–qPCR. Complementary DNA (cDNA) from whole mammary gland cells was synthesized from total RNA (1 μg) using PrimeScript Reverse Transcriptase Kit. PCR amplification was performed by using Power Sybr Green, and fluorescence was analyzed with AriaMx Real‐Time PCR System. The 2^−ΔΔCt^ method was used to determine the relative abundances of mRNA in each experimental condition using *gapdH* as endogenous control and normalizing to the values obtained in the PBS group as follows:

ΔCt = Mean Ct analyzed gene − Mean Ct *gapdH*
ΔΔCt = ΔCt treated group − ΔCt PBS group
Relative abundance = 2^−ΔΔCt^

Primer pairs for *gapdH* (Regueiro *et al*, 2011), *tnf‐α*, *kc (il‐8*), *mip‐1a*, *mcp‐1*, *inf‐γ*, *tlr2*, *il‐1β*, *il‐4*, *il‐6*, *il‐12p40*, *il‐18*, and *il‐23* detection are shown in Dataset EV7.

#### Subcutaneous inoculation of Mycoplasma pneumoniae strains in CD1 mice

To study the adaptive immune response, thirty CD1 female mice (4–5 weeks old) were subcutaneously inoculated with one (day 0) or two doses (day 0 and day 4) of a 100 μl of a bacterial solution containing ˜10^8^ CFU/mouse of a WT or CV2 strain, using a 25G syringe. On day 18, mice were anesthetized (isoflurane 2%) for intracardiac blood extraction (˜1 ml/mouse) and immediately euthanized by cervical dislocation. Blood was collected in sterile Eppendorf tubes and maintained at room temperature until a clot appeared. Tubes were centrifuged 15 min at 3,787 *g*, and the resulting supernatant was stored at –80°C until use. For macroscopic evaluation of tissue injuries, subcutaneous tissue was dissected. The control group was inoculated with the same volume of vehicle solution (PBS 1×) and processed in parallel.

#### *Measurement of INF‐y*, *IL‐4*, *IgM*, *and IgG in serum*

Concentrations of INF‐γ, IL‐4, IgM, and IgG proteins were measured by ELISA in serum samples, following the manufacturer’s instructions. Their levels of sensitivity were as follows: INF‐γ, 15.6 pg/ml; IL‐4, 2.0 pg/ml; IgM, 2.53 Novatin units (NU)/ml; and IgG, 2.67 NU/ml. For IgM and IgG determination, NU were calculated as follows:

NU = (absorbance value × 10)/cut‐off.

Interpretation of results: cutoff value, NU = 10; positive: NU > 11; gray zone, NU = 9–11; negative, NU < 9. All samples were run as duplicates.

#### Mycoplasma pneumoniae doubling times determination

Pre‐quantified stocks of the different strains were grown in T25 flasks for 48 h using as starting inoculum 5 μg of protein as determined by Bicinchoninic acid (BCA) assay. To treat all the strains (i.e., adherent and non‐adherent) similarly, cells were collected in the media in which they were growing and subjected to two rounds of centrifugation (15,000× *g*, 5 min) and washing in PBS before resuspending the cell pellet in lysis buffer (10 mM Tris, pH 8.6, 6 mM MgCl_2_, 1 mM EDTA, 100 mM NaCl, 0.1% Triton X‐100 plus a cocktail of protease inhibitors). Subsequently, the total protein content of the cultures was determined by BCA assay, and the doubling times of each strain estimated as follows:

Growth rate (μ) = ln (final protein / initial protein) / 48 h
Doubling time = ln (2) / μ

#### Mycoplasma pneumoniae secretome and proteome characterization

For secretome samples, *M. pneumoniae* is usually cultured in modified Hayflick media, a rich media containing many proteins from added horse serum. However, the highly abundant proteins from the rich media covered the signal from low abundant secreted proteins, leaving them unsuitable for mass spectrometric (MS) analysis. Therefore, we used the minimal media of *M. pneumoniae* as growth media for our experiment (Yus *et al*, 2009). This minimal media still contains bovine serum albumin (BSA) as lipid carrier in high amounts. We replaced BSA with 5 mM (2‐hydroxy)propyl‐β‐cyclodextrin (Hyprop) (Greenberg‐Ofrath *et al*, 1993) to obtain a protein‐free media compatible with downstream MS analysis.

For the secretome samples, the WT M129 strain was grown in Hayflick rich media for 3 days. The cultures were washed twice with PBS while attached, and twice after scraping and centrifugation at 21,000 *g*. Cells were then split 1:10 into two different 150‐cm^2^ flasks containing 40 ml of Hyprop media. Cells of both flasks were allowed to attach for 24 h and then washed twice with PBS to remove all possible trace amounts from the horse serum coming from Hayflick media. After this, both flasks were filled again with 40 ml of Hyprop media and incubated at 37°C for 72 h. Next, the culture supernatant and the cells of one the flasks were collected (representing the 72‐h secretion time point), whereas the supernatant of the remaining flask was discarded, and the attached cells were washed twice with PBS and incubated for additional 24 h with 40 ml of fresh Hyprop media (representing the 24‐h secretion time point) before being processed in the same way as the previous flask. For both flasks, cells were scraped in the same volume of Hyprop media as the collected supernatant. Cell suspensions were processed identically and in parallel to the supernatants, to avoid any bias from experimental procedure in the outcome.

Samples were precipitated with 60% acetone and 10% trichloroacetic acid (TCA) as final concentration. The mixture was spun for 1 h at 35,000 *g* (4°C). The supernatant was discarded, and the pellets were resuspended in 1.5 ml of TCA/acetone and spun 2 h at 16,000× *g* (4°C). The supernatant was removed and the pellet was dried completely in a speed vac before being resuspended in a buffer of 8 M urea and 100 mM NaHCO_3_, using a bioruptor system. The total protein amounts in the samples were determined using BCA assay. The UPF‐CRG Proteomics facility digested and labeled the samples. Primary amines (i.e., the N‐terminus and the ε‐amino group of lysine) of the proteins were tagged using dimethyl labeling. Specifically, the extracts were labeled with isotopes differing in their mass numbers (e.g., heavy and light isotopes). Equal amounts of the different samples were then mixed for a given condition (e.g., extracellular and intracellular extracts of a particular time point) and the areas under the curves of the tryptic peptides were determined by nanoLC/MS/MS. The ratios of the same peptides containing different isotopes (corresponding to intracellular and extracellular protein extracts) were calculated as previously described (Tolonen *et al*, 2011). The *P* values of a bimodal distribution were calculated by standard methods, and a conservative *P*‐value of 0.001 was employed as a threshold to define a protein as secreted.

For the proteome samples of different mutant strains, mycoplasma strains were grown at an exponential phase of growth. Then, the medium was removed and cells were washed twice with PBS. Total protein extracts were obtained by lysing the cells with 200 µl of lysis buffer (4% SDS, 0.1 M DTT and 0.1 M Hepes). The total protein extracts of two biological replicates were analyzed by LC/MS/MS. Briefly, samples were dissolved in 6 M urea, reduced with 10 mM dithiothreitol (37°C, 60 min), and alkylated with 20 mM iodoacetamide (25°C, 30 min). Samples were diluted 6‐fold with 0.2 M NH_4_HCO_3_ before being digested at 37°C overnight with trypsin (ratio protein:enzyme 10:1). Peptides generated upon digestion were desalted, evaporated to dryness and dissolved in 0.1% formic acid. An aliquot of 2.5 µl of each fraction (amounts ranging from 0.17 to 4 µg) was run on an LTQ‐Orbitrap Velos fitted with a nanospray source after a nanoLC separation in an EasyLC system. Peptides were separated in a reverse phase column, 75 μm × 250 mm with a gradient of 5–35% acetonitrile in 0.1% formic acid for 60 min, at a flow rate of 0.3 ml/min. The Orbitrap Velos was operated in positive ion mode with the nanospray voltage set at 2.2 kV and its source temperature at 325°C. In addition, 20 µg of the total extract was digested and desalted, and 1 µg of the resulting peptides was analyzed on an Orbitrap Velos Pro using the same conditions as the fractions but with a longer gradient (120 min). Two technical replicates for each strain were analyzed unless otherwise indicated. The spectra were assigned to peptides by using Mascot and a customized database comprising all the ORFs longer than 19 amino acids. Protein abundance was estimated as the average area under the height of the precursor ions for the three most abundant peptides of each protein. In order to correct for systematic differences in the distribution of protein abundances between samples, summarized protein areas were normalized by the median of the areas distribution for this sample. The log2 of the normalized area fold‐changes between mutant strains and the WT strain was computed. Significant changes in protein abundance were assessed by independent two‐sided *t*‐test (scipy.stats python package v1.5.0 (Virtanen *et al*, 2020)), followed by multiple tests correction using Benjamini‐Hochberg method with 5% family‐wise false discovery rate (Benjamini & Hochberg, 1995). This analysis was restricted to those cases in which protein values from two technical replicates were available in all the strains, and no significant changes were found in the abundances of any protein compared to those observed in WT strain.

#### Validation of secretory signals with Alginate Lyase

Alginate lyase activity in the supernatants of the engineered strains was determined following a turbidimetric assay previously reported (Kitamikado *et al*, 1990). Briefly, at the indicated time points after culture inoculation, samples of the supernatants (200 µl) were collected and mixed with 2 ml of acidic albumin solution (3.26 g sodium acetate, 4.56 ml glacial acetic acid, 1 g bovine albumin fraction V, filled up to 1 l with water and adjusted to pH = 3.75 with HCl). A white precipitate is formed in the presence of polymeric alginate. Thus, the presence of alginate can be determined after transferring 200 µl of the mixture to a multiwell plate and measuring OD at 300 nm.

#### In vitro antimicrobial activity on S. aureus growth curves

Cultures of *M. pneumoniae* strains expressing lysostaphin under different promoters were grown in 25‐cm^2^ flasks filled with 4 ml of Hayflick medium. After 3 days, 1 ml of the culture supernatant was collected and passed through a 0.22‐µm filter.

For *S. aureus*, an overnight culture was diluted to OD 600 nm of 0.032. Then, 180 µl of this starting culture was added to each well of the 96‐well plate and its growth (at 37°C with 1,080 rpm continuous agitation) was followed by measuring OD 600 nm every 20 min in a TECAN plate reader. After 6 h, 20 µl of different mycoplasma samples (described above) or of recombinant lysostaphin stocks with different concentrations was added to the well. *Staphylococcus aureus* growth was tracked for an additional 9 h after treatments.

#### Dispersal assays of biofilms performed in vitro in multiwell plates

An overnight *S. aureus* culture was diluted 1:40 in TSB‐glc and dispensed (100 µl/well) in 96‐well polystyrene microtiter plates. Plates were incubated at 37°C for 24 h to obtain mature biofilms and washed with Hayflick to remove free cells. The preformed biofilms were treated (100 µl/well) with WT, WT‐DispB, CV2, or CV2‐DispB cell suspensions containing ≈1 × 10^9^ CFU/ml or a similar volume of the culture supernatants of these strains filtered by 0.2‐µm filters. As a control to normalize all the treatments, some wells were treated with Hayflick. Also, a staining control was carried out in wells in which no *S. aureus* biofilms were formed. After 15 min, 4, 8, or 24 h of incubation at 37°C, wells were stained with crystal violet 0.1% (15 min, room temperature), washed, and air‐dried. The crystal violet attached to the biofilm was solubilized with 100 µl/well of ethanol:acetone (80:20, vol/vol) and quantified by reading the absorbance at 595 nm (OD 595 nm) in a Multiscan microplate reader.

#### In vitro dispersal assay of catheter‐associated biofilms

Sealed catheters and *S. aureus* mature biofilms were prepared as previously reported (Garrido *et al*, 2014). Briefly, commercial Vialon^®^ 18G 1.3‐ by 30‐mm catheters were cut into 20‐mm segments and sealed under sterile conditions with petrolatum and tissue glue. Cleaning and disinfection were achieved thereafter by immersion in DD445 and ethanol (15 min in each solvent). Sterility of catheters was checked by absence of turbidity after 24‐h incubation (37°C) in TSB. To establish mature biofilms, sterile catheters were immersed in 6‐well polystyrene plates containing 1 ml of a suspension containing ≈1 × 10^6^ CFU of *S. aureus* in TSB‐glc and incubated at 37°C for 24 h. After that, catheters were rinsed with 1 ml of Hayflick medium and treated for 4 h, at 37°C, with 1 ml of ≈1 × 10^8^ CFU of mycoplasma cells. The biofilms attached to the catheters were stained with crystal violet 0.1% (15 min, room temperature) and subsequently destained with ethanol:acetone (80:20, vol/vol). The resulting solution was quantified by OD 595 nm in a Multiscan microplate reader. As a staining control, catheters non‐infected with *S. aureus* were treated with 1 ml of Hayflick and processed in a similar manner.

#### Ex vivo dispersal assay of catheter‐associated biofilms

Catheters carrying *S. aureus* biofilms were generated as described above and immediately implanted subcutaneously in anesthetized mice through a minimal surgical incision in the interscapular area. After 18 h, animals were euthanized to remove aseptically the catheters, which were individually rinsed with PBS, treated (37°C, 4 h) with 1 ml of 1 × 10^8^ CFU of mycoplasma cells and processed as described above. Also, a staining control based on catheters non‐infected with *S. aureus* was included.

#### In vivo evaluation of catheter‐associated biofilms treatment by [^18^F]‐FDG‐Micro‐PET

To monitor infection by [^18^F]‐FDG‐MicroPET imaging, as previously detailed (Garrido *et al*, 2014), fasted mice were anesthetized with 2% isoflurane in O_2_ gas inhalation, and intravenously injected with 18.8–1.9 MBq of [^18^F]‐FDG. After 1 h of radiotracer uptake under continuous anesthesia, PET images were taken in a small‐animal tomography apparatus (MicroPET) by laying mice in a prone position and capturing images for 15 min. Images were reconstructed using a true three‐dimensional (3D) Ramla algorithm reconstruction with 2 iterations and a relaxation parameter of 0.024 into a 128 by 128 matrix with a 1‐mm voxel size, applying dead time, decay, random, and scattering corrections. For [^18^F]‐FDG uptake assessment, MicroPET images were analyzed using the PMOD software, and semiquantitative results were expressed as the standardized uptake value (SUV) index, obtained by normalization with the formula SUV = [(RTA/cm^3^)/RID] × BW, where RTA is the radiotracer tissue activity (in becquerels), RID is the radiotracer injected dose (in Bq), and BW is the mouse body weight (in grams). After qualitative inspection of the images, volumes of interest (VOI) were manually drawn on coronal 1‐mm‐thick consecutive slices including the entire catheter area. For catheter image quantification, to avoid manual bias of surrounding areas, a new VOI was generated semi‐automatically using the threshold of 60% of maximum pixel for SUV mean calculation (SUV60 index). The results were expressed as the % of SUV 60 increase calculated as follows: [(SUV60 at day 4 × 100) / SUV60 at day 1] ‐ 100.

## Author contributions

VG designed and performed the animal studies; CP‐L designed, obtained, and characterized the mycoplasma strains and wrote the initial draft of the manuscript; IR‐A evaluated the adaptative immune response and the cytokine profiles by RT–qPCR; BP did the analysis of the secretome; TF analyzed the antibiofilm *in vitro* activity of the different enzymes expressed in *M. pneumoniae*; MW assembled the genome of CV2 strain, EG‐R and CG determined the growth parameters of mycoplasma strains, MC and IP designed and did the PET studies in mice; LS, M‐JG, and ML‐S conceived the work and supervised the work of the team; all authors discuss the results and revised the manuscript; ML‐S and CP‐L coordinated the redaction of the final manuscript.

## Conflict of interest

The work described here was done before the creation of the start‐up Pulmobiotics. In any case, we would like to state that Carlos Piñero‐Lambea and Maria Lluch‐Senar are now working in Pulmobiotics Company and Luis Serrano and Maria Lluch‐Senar are co‐founders of the company. This company is interested in the development of *M. pneumoniae* as a vector to treat human lung diseases. There are three patents protecting the results shown in the current work (EP 16706622.4; EP20382208.5 and EP20382288).

## Supporting information



Expanded View Figures PDF

Dataset EV1

Dataset EV2

Dataset EV3

Dataset EV4

Dataset EV5

Dataset EV6

Dataset EV7

## Data Availability

Raw data of mass spectroscopy spectra are deposited at PRIDE database with the project accession: PXD028011 (http://www.ebi.ac.uk/pride/archive/projects/PXD028011). The complete sequence of the most relevant plasmids designed in this study as well as the genome assembly of CV2 strain can be found at the European Nucleotide Archive (ENA) under the study accession number PRJEB45050 (http://www.ebi.ac.uk/ena/data/view/PRJEB45050).
