# Supplementary material for: Engineering a genome‐reduced bacterium to eliminate Staphylococcus aureus biofilms in vivo
Source: Mol Syst Biol. 2021 Oct 6;17(10):e10145. doi: 10.15252/msb.202010145 (PMC8493563; doi:10.15252/msb.202010145)
Supplement: Supplementary file 1 — Expanded View Figures PDF [file MSB-17-e10145-s003.pdf]

Expanded View Figures

Figure EV1. Generation and characterization of *M. pneumoniae* mutant strains.

A Scheme depicting the protocol followed for the generation of ssDNA recombineering substrates employed to obtain the engineered strains. The illustration shows (from left to right) the amplification of dsDNA PCR products, their incubation with streptavidin-coated magnetic beads, the NaOH-mediated release of the strand of interest and the electrophoresis analysis of the products before and after ssDNA purification.

B Top, electrophoresis analysis of the PCR products obtained at each edited loci for the indicated strains. The internal code of the oligos employed for the screening is shown in brackets, and their sequences can be found in Dataset EV6. Bottom, scheme depicting the expected sizes of the PCR products if the respective locus is edited or not.

C Plot showing the results of the mass spectrometry analysis done for the mutant strains generated in this work. Bars represent the area under the curve (AUC) values for the three most abundant peptides of each protein in the proteome. Results are shown as the mean ± SD of two technical replicates ($n = 2$), except for Δ*mpn453* strain for which only data for one technical replicate are available. The complete data of the MS analysis can be found in the Dataset EV1. Note that the Δ*mpn051* strain was not included in this analysis, as its corresponding edited gene is disrupted by a transposon insertion, and not deleted.

D Plot showing the estimated doubling times of the mutant strains after 48 h of growth calculated by total protein content increase. Results are shown as the mean ± SD of three biological replicates ($n = 3$). Complete data of this analysis can be found in Dataset EV2. Results from Fisher's PLSD test are shown for those strains that showed a doubling time statistically different from that of the parental WT*gp35* strain. ***$P \leq 0.0005$; ****$P \leq 0.00005$.

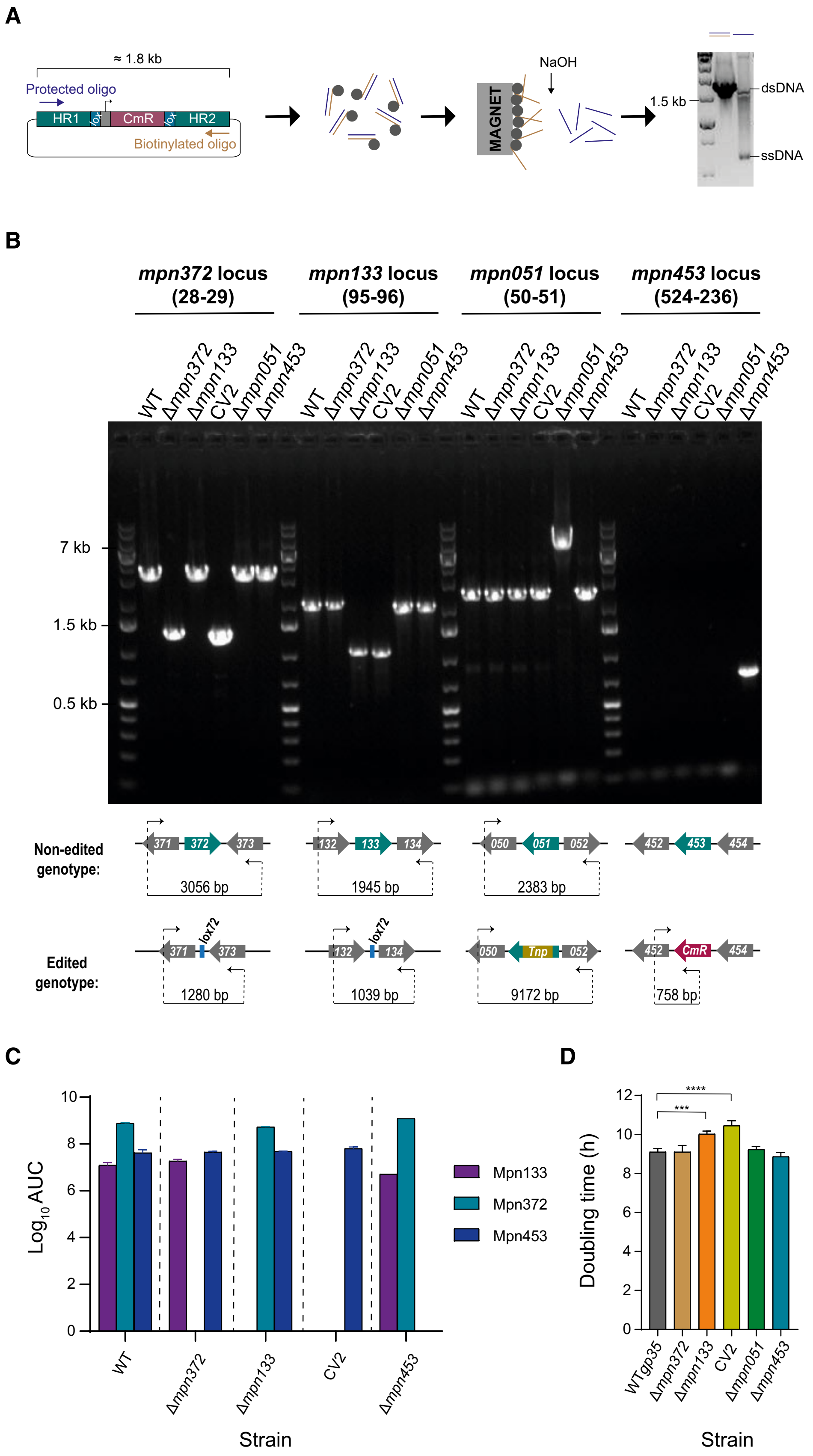

Figure EV1.

© 2021 The Authors

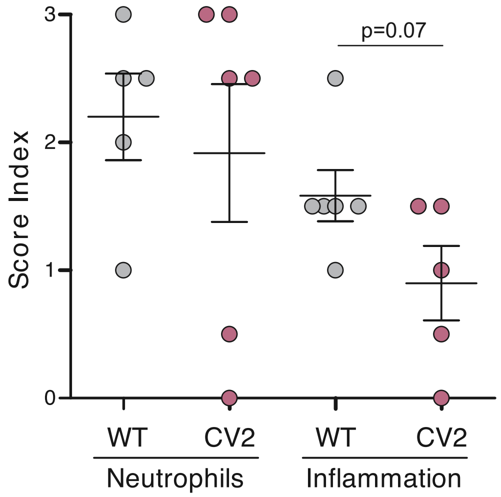

Figure EV2. Histological score of mouse mammary glands infected with *M. pneumoniae* WT and CV2 strains.

Plot showing the results of the blind histopathological analysis carried out at 4 days post-infection of the abdominal mouse mammary glands. Samples were excised and processed by hematoxylin-eosin staining and scored (0–3) for the presence of neutrophil infiltration and interstitial inflammation. Each circle represents the scored assigned in individual samples ($n \geq 5$), whereas mean ± SD is represented with lines inside each group. No statistical differences were found between infection groups by Fisher's PLSD test, although a strong statistical tendency ($P = 0.07$) was found for interstitial inflammation as shown in the graph.

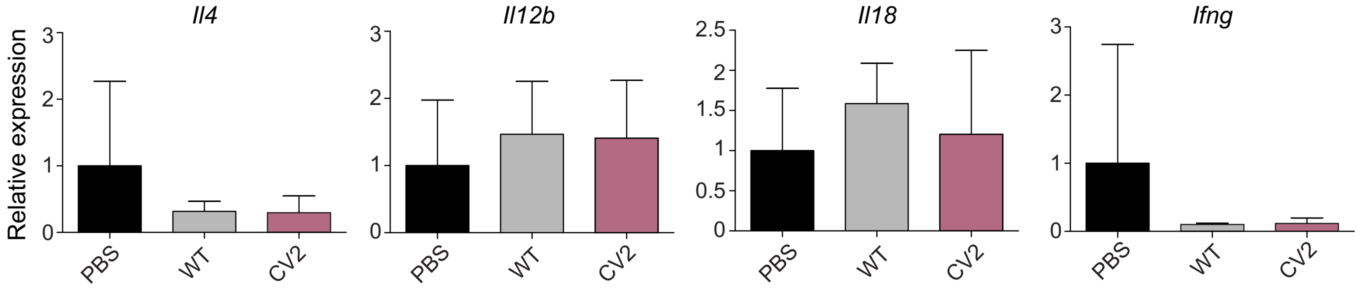

Figure EV3. Expression profile of other interleukins in animals treated with CV2 or WT strains.

Plots showing the RT–qPCR analysis conducted to quantify relative expression of the indicated interleukins coding genes in the mammary glands. Results are expressed as mean ± SD ($n = 10$ for PBS, $n = 5$ for WT, and $n = 4$ for CV2) of the $2^{-\Delta\Delta Ct}$ relative expression values of the indicated interleukins; the value from each individual animal was calculated from three technical replicates. The values obtained in the PBS group were used as control for normalization of gene expression (= 1). Statistical analysis was performed using a one-sided ANOVA followed by the post-hoc Fisher's PLSD test. No statistically significant differences were found between the different groups.

© 2021 The Authors

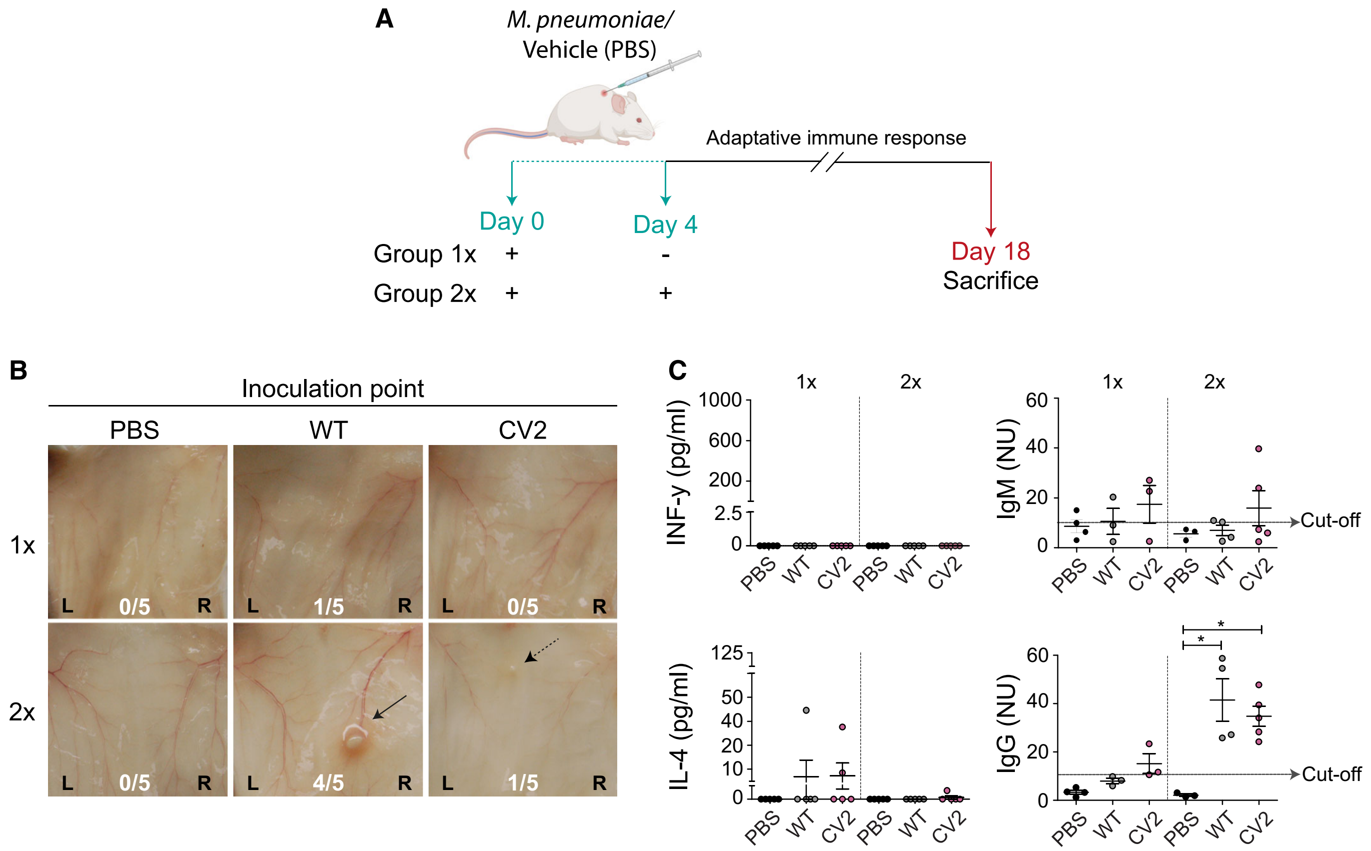

Figure EV4. Evaluation of adaptive immune response induced by *M. pneumoniae* strains in a subcutaneous mice model.

A Experimental design. CD1 female mice were inoculated subcutaneously with a single or repeated bacterial solution of WT or CV2 containing 1×10^8 CFU/mouse at day 0 or day 0+ day 4 (referred to as group 1× and group 2×, respectively). On day 18, animals were sacrificed. The image was created with the help of BioRender.com.

B Macroscopic evaluation of the subcutaneous tissue of animals subjected to one (1×) or two doses (2×) of PBS, WT or CV2. The ratio of animals showing relevant findings at the inoculation point is shown within each picture (L, left; R, right).

C Determination of INF-γ, IL-4, IgM, and IgG protein levels in serum samples measured by ELISA. Each circle represents the values obtained in individual animals ($n \geq 3$), subjected to one (1×) or two doses (2×), whereas mean ± SD is represented with lines inside each group. Statistical analysis was performed using one-way ANOVA and the Tukey post-hoc test. $^*P < 0.05$.

© 2021 The Authors

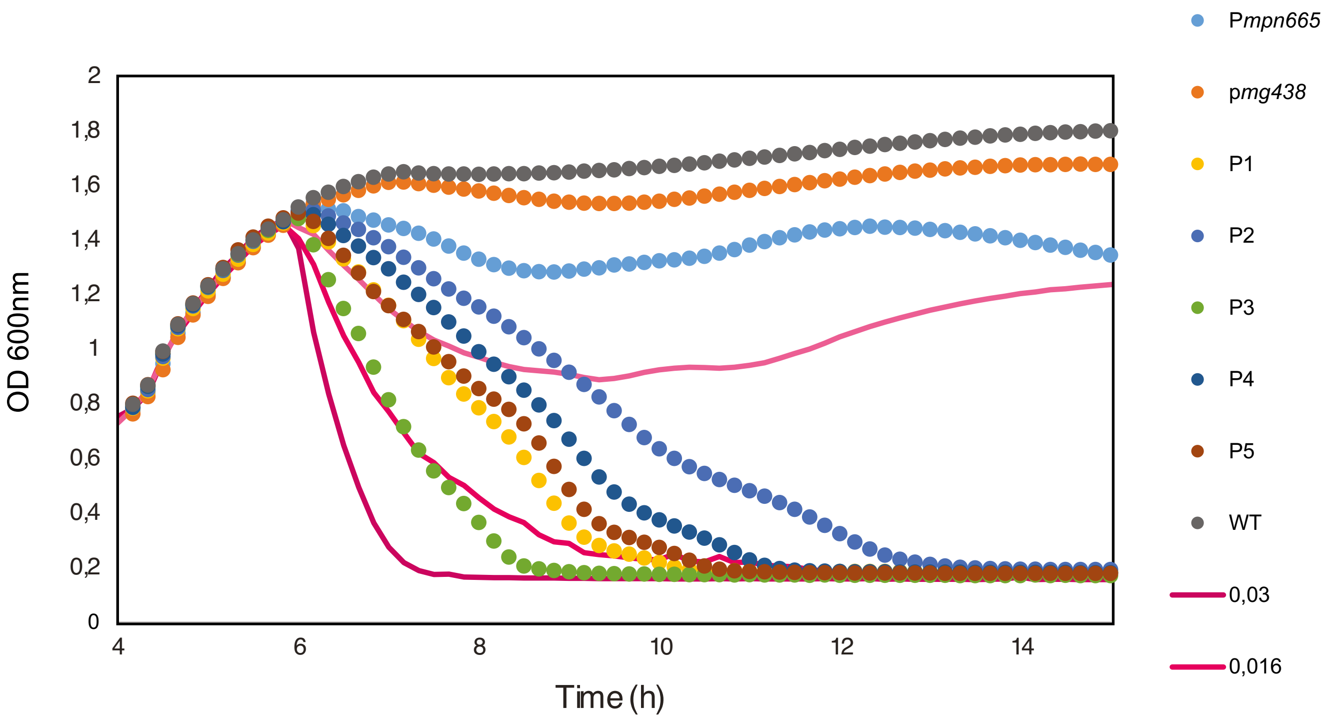

Figure EV5. Impact in *S. aureus* growth curves of lysostaphin production by *M. pneumoniae* driven by different promoter sequences.

Plot showing a growth curve of *S. aureus*. After 6 h of growth, 20 µl of different treatments was added. Dotted lines represent treatments based on culture supernatants ($n = 1$) of different *M. pneumoniae* strains carrying the *mpn142Opt*-Lysostaphin construct under control of the indicated promoter sequences. A treatment based on the supernatant of a culture of WT strain was added as control. Continuous lines represent treatments based on recombinant lysostaphin protein at the indicated concentrations in µg/µl.

© 2021 The Authors
