# Supplementary material for: Engineering a genome‐reduced bacterium to eliminate Staphylococcus aureus biofilms in vivo
Source: Mol Syst Biol. 2021 Oct 6;17(10):e10145. doi: 10.15252/msb.202010145 (PMC8493563; doi:10.15252/msb.202010145)
Supplement: Supplementary file 9 [file MSB-17-e10145-s002.pdf]

molecular systems biology

Engineering a genome-reduced bacterium to eliminate Staphylococcus aureus biofilms in vivo

Victoria Garrido, Carlos Piñero-Lambea, Irene Rodriguez, Bernhard Paetzold, Tony Ferrar, Marc Weber, Eva Garcia-Ramallo, Carolina Gallo, Maria Collantes, Iván Peñuelas, Luis Serrano, María-Jesús Grilló, and Maria Lluch-Senar
DOI: 10.15252/msb.202010145

Corresponding author(s): Maria Lluch-Senar (maria.lluch@pulmobio.com) , Luis Serrano (luis.serrano@crg.eu), María-Jesús Grilló (mj.grillo@csic.es)

Review Timeline:

Submission Date:	1st Dec 20
Editorial Decision:	7th Jan 21
Revision Received:	25th Jun 21
Editorial Decision:	15th Jul 21
Revision Received:	14th Sep 21
Accepted:	15th Sep 21

Editor: Maria Polychronidou

Transaction Report:

(Note: With the exception of the correction of typographical or spelling errors that could be a source of ambiguity, letters and reports are not edited. Depending on transfer agreements, referee reports obtained elsewhere may or may not be included in this compilation. Referee reports are anonymous unless the Referee chooses to sign their reports.)

Thank you again for submitting your work to Molecular Systems Biology. We have now heard back from the three referees who agreed to evaluate your study. Overall, the reviewers think that the study presents a relevant contribution in the context of engineering therapeutic bacteria. However, they raise a series of concerns, which we would ask you to address in a major revision.

The reviewers' recommendations are rather clear and I think that there is no need to repeat all the points listed below. All issues raised by the reviewers would need to be convincingly addressed. Please let me know in case you would like to discuss any of the issues raised. As you might already know, our editorial policy allows in principle a single round of major revision so it is essential to provide responses to the reviewers' comments that are as complete as possible.

On a more editorial level, we would ask you to address the following issues.

REFEREE REPORTS

Reviewer #1:

Engineered bacteria have emerged as a promising platform for delivering therapeutic payloads in a targeted, sustained, and well-regulated manner. Criteria for selecting an appropriate bacterial chassis depends on the disease context: the engineered bacteria should be viable in the anatomical location being targeted and possess the metabolic capability to express the desired

therapeutic payload. Hence, expanding our repertoire of engineerable bacteria will facilitate efforts targeting a wide array of disease.

In this study, Garrido et al. engineer an attenuated strain of Mycoplasma pnemoniae to eradicate Staphylococcus aureus biofilms. The authors knockout genes contributing to M. pnemoniae virulence in order to create an attenuated strain that is amenable to non-pathological in vivo administration. This attenuated strain is engineered to secrete Dispersin B, which demonstrates efficacy in dispersing S. aureus biofilms both in vitro and ex vivo. When the attenuated M. pnemoniae strain secreting Dispersin B does not effectively disperse S. aureus biofilms in vivo, the authors additionally include a gene for secreting Lysostaphin which consequentially improves biofilm eradication.

While the concept of using engineered bacteria to eradicate biofilm-forming pathogens is not novel (ref. 61), the study nonetheless provides advances in i) engineering a "safe" genome-reduced bacterium for therapeutic function and ii) using engineered bacteria to clear subcutaneous infections caused by biofilm-forming pathogens. However, there are a number of issues the authors should address before the work is suitable for publication:

Major comments
1) The manuscript tends to highlight results obtained using WT and WT-DisB, which are the pathological strains. The figures and text should instead emphasize results obtained from the CV2 and CV2-DisB strains, as these are the non-pathological strains that would actually be used as a therapeutic. In particular, Fig. 3A should show a time-course for the CV2 and CV2-DisB strains (instead of WT and WT-DisB), and results section #5 paragraph #2 should reflect this change.

2) For in vivo studies, the authors use PET scans to track S. aureus biofilm production. While this technique allows comparisons between different treatment groups and facilitates longitudinal examination, it does not provide specific information about the absolute change in S. aureus abundance or biofilm production. Instead, this PET signal is seemingly dependent on a number of factors including bacterial abundance and inflammation (ref. 43). Ideally the authors would also provide CFU counts to demonstrate absolute changes in S. aureus abundance or discuss how PET signal corresponds to absolute S. aureus abundance. For example, what does a 20% increase or decrease in PET signal correspond to in terms of S. aureus abundance?

3) The authors claim that the attenuated CV2 strain is non-pathological; however, it seemingly still elicits an immune response (Fig. S1). The authors should address whether the fact that the CV2 strain still elicits a weak immune response precludes it from long-term or repeated use. For example, is there evidence that no adaptive immune response will be mounted against the attenuated strain?

4) The final demonstration (Fig. 5D) using the CV2-DisB-Lyso strain performs inconsistently in vivo (4/11 replicates showed no decrease in S. aureus loads). A clear explanation of how this is an improvement over existing methods therapies is warranted. Additionally, the authors should discuss improvements that would facilitate the use of CV2-DisB-Lys as a more robust therapy.

Minor comments
1) The authors offer no definition or explanation of the standardized uptake value (SUV), except for a mathematical equation in the supplement. The results reported in Fig. 5 would be more accessible to a broader audience if the authors qualitatively defined the SUV60 in the main text and described what the SUV measures (i.e. bacterial abundance and inflammation).

2) The authors should comment on biocontainment characteristics for M. pnemoniae. For example, how long is M. pnemoniae expected to colonize? Ideally M. pnemoniae will only be present throughout the therapeutic course and will not persist in the body.

3) The authors allude to the fact that S. aureus growth curves are used to assess efficacy of Lysostaphin-killing. However, these growth curves do not appear to be in the main text or supplementary files. These growth curves should be included to demonstrate the effect CV2-DisB-Lys has on S. aureus growth.

4) In addition to treating biofilms with systemically administered biomolecules, there have been growing advances in using phage to treat biofilms (Khalifa et al. (2015) Applied and Environmental Microbiology; Lu et al. (2007) PNAS). The authors should acknowledge some of these efforts and discuss the advantages of using bacterial therapies.

5) The authors claim, "No significant changes were found in the doubling times of the generated strains ..." but do not provides statistical analyses to validate their use of "significant". Similarly, the authors state: "... the cytotoxic nuclease (mpn133) and CARDS toxin (mpn372) contribute to M. pneumoniae virulence; and have a synergistic effect" yet do not provide any quantitative analyses to validate their use of "synergistic". The authors should either provide quantitative support for this terminology or replace these terms.

6) At the end of the introduction, the authors introduce the term "Mycochassis" to denote their attenuated strain. However, this term is never used again. We recommend removing the term "Mycochassis".

7) The authors claim, "data for mpn133 and mpn051 mutants was obtained from previous work" - this previous work needs to be cited.

8) The order of conditions in Fig. 1C should be the same as in Fig. 1A. Currently, the delta mpn372 and delta mpn133 are not in the same order.

9) The authors should remove "beautifully" in the following sentence to help omit flowery and unnecessary language: "This is beautifully exemplified in this work by the comparison between cell and supernatant treatments."

10) There is a typo in the methods: "A detailed list of plasmids and the sequence of the different modules included on them is available on Table S6." Table S6 contains the qPCR primers and not the plasmid sequences.

11) The scale bar in Fig. 5 should have a title (e.g., "SUV60).

12) The authors should provide sequences for the 5'UTR and 3'UTR regions used to express proteins, such that the work can be reproduced or adapted.

13) There are a couple of sentences that are difficult to understand and need to be reworded. I've listed them below:
- "Moreover, the absence of cell wall also facilitates direct release of secreted bioactive compounds, which might be combined when required with antibiotics targeting cell wall formation without affecting the viability of the therapeutic Mycoplasma strain."

- "As up to 90% of the total biofilm thickness can be attributed to this matrix, S. aureus cells encounter in
these structures a great protection against antimicrobial agents and host defense mechanisms."

Reviewer #2:

Garrido et colleagues report the creation of attenuated Mycoplasma pneumoniae strains as live biotherapeutic chassis to treat bacterial infections. They first show that deleting selected genes encoding virulence factors results in colonization of the mouse mammary gland without causing macroscopic lesions. The authors also investigate the production of cytokines by the mammary gland tissue by RT-qPCR. The next step was to perform mass spectrometry and bioinformatics analyses to identify secreted proteins along with their respective signal peptides. Using the mpn142 signal peptide, dispersine B was introduced in wild-type or attenuated (CV2) M. pneumoniae chasses, and their ability to disrupt biofilms was assayed in vitro and ex vivo. Finally, the authors performed an in vivo assay using a mouse model of catheter infection. The results indicate that the attenuated chassis was not as efficient as the wild-type in this context, leading to an experiment where both the WT and CV2 strains were combined. A CV2 strain producing dispersine B and lysostaphin also generated better results than CV2 secreting dispersine B alone.

Globally, I found the rationale of the project interesting and I believe there is an expanding readership for this type of work To my knowledge, there is no other group engineering mycoplasma species as living medicines. Using a near-minimal well less bacterium as a live biotherapeutics could indeed provide a new approach to fight bacterial pathogens. However, many aspects are difficult to understand and there is an important "lack of finish" that decreases the overall quality of the manuscript. There are many typos, errors, sentence the are missing a few words or details that are not clearly explained. The manuscript thus appears to lack important information needed to appreciate the data and the overall work. The authors should also be careful about the wording of some claims. I am detailing some of these issues below.

---MAJOR COMMENTS---

-Rewording would be required to tone down certain claims in the title and abstract:
o The title can be misleading. M. pneumoniae has a naturally small genome but the chassis presented here is not minimal per see.
o Abstract: M. pneumoniae does recombine. There is evidence of recombination in the literature (e.g. PMID: 20543037). The introduction offers more nuance about this but the abstract is misleading.
o Abstract: The genetic code does not prevent potential genetic exchanges between mycoplasma.

-Several genes were deleted to obtain an attenuated strain of M. pneumoniae. The phenotypes of the resulting strains were analyzed by mass spectrometry. Why not sequence the genomes of the strains to confirm these deletion events? Was this done? The wording suggests that genotyping was not performed. Mass spectrometry alone could be misleading as it is possible that there would be no expression under those laboratory conditions.

- The authors state that there were no significant changes in the doubling times of the various

mutant strains. How can we be sure about this as there is no growth curve presented and there are no standard deviations presented with the data? There is almost a two-hour difference between the slowest- and the fastest-growing mutant strains.

-The immune response of the mammary gland tissue was investigated by qPCR. Although other assays may arguably offer a better assessment of the effective response, I understand that RT-qPCR is a more accessible approach to answer this question. However, I doubt that the comparative threshold cycle (Ct) method was applied correctly. This method generally uses the is usually 2 (or whatever the reaction efficiency is) to the power of the delta-delta Ct, and the result should be a fold change. The equation presented here seems to include only a delta Ct and is multiplied by 100, which results in some cases in very low numbers (between 0.05 and 0.5 with large error bars in the case of IL-6). There is no reference for the method that was applied. This should be clarified or calculated with a more standard approach.

-The section about the identification of the secretion signals (section number 4) is very difficult to follow, in part because Table S3 is not clearly presented. How is the table sorted and why (see below for additional points on supplementary data)? It is thus hard to understand what proteins the authors refer to when mentioning:11 proteins of which 6 were in the top 20 and 4 were predicted to be membrane proteins. The authors also indicate in the text that 3-4 residues were added in addition to the predicted signal peptides but Table 1 rather mentions 5 residues (and these residues are not clearly indicated in Table 1). The strategy and results should be easier to follow.

-It looks like some data is missing from the manuscript about S. aureus growth curves (1st line of 4th paragraph of Results section 7): "Then, we checked the bacteriolytic activity of different strains on S. aureus growth curves"; Where are these results? This is pretty important since the next sentence indicates that "strains with endogenous promoters showed minor effects on S. aureus growth curve"... What about strains with P1-P5 promoters? Did they show any antimicrobial effect?

-The supplementary information is not clearly presented.

-Table S1:
o No context to understand the information presented in the first line
o What is the difference between an empty cell and a value of zero?
o What are the last three columns?
o Why use the area under the curve for the 3 most abundant peptides instead of normalized spectral abundance?

-Table S2:
o No growth curve is shown in the manuscript; these are growth parameters.
o How qPCR measurements described in the methods were converted into biomass?
o How can we know if there is a significant difference in the doubling time if there is no standard deviation?

-Table S3:
o How is this table sorted? This is extremely difficult to understand with the current explanations.
o There are two T1-2 columns for the p-value
o What is the difference between an empty cell and a value of zero?

-Figure S1: how is the score defined? Was this performed in a double-blind manner? This type of

subjective data should be blinded.
o How can we know if there is a significant difference in the doubling time if there is no standard deviation?

-Figure S3:
o there is no title on the x-axis. One can deduce that these are gene numbers for the signal peptide but this should be explicit in the figure or the figure legend.

---MINOR COMMENTS---

There are several typos, missing words, etc. throughout the documents that make the work more difficult to appreciate.

-Figure 1A: give the +++ / - lesion score in A instead of C ? Why are the gene deletions not presented in the same order between panel A and panel C? Also, why give the p-value thresholds in legend if no difference was observed?

- Figure 2 and Legend Figure 2: 2-Ct*100 (legend) and 2Ctx100 (figure)? I do not understand these units. Normally, we provide RT-qPCR results as relative expression values (fold change) using the 2^-Ct formula, and arbitrarily setting the control group to a value of 1 (PBS). Also correct Figure S2 and methods accordingly.

- Mass spectrometry: why use area under the curve for the 3 most abundant peptides instead of peptide count / of NSAF?

- The results section describing the construction of transmembrane proteins fused to the alginate lyase protein (section 4) is very difficult to understand clearly. Please revise the text as many errors and syntax problems are present. A figure summarizing the design would also greatly help the comprehension of this section.

- italicize in vivo in title and abstract as for the rest of the paper. Gene names should also be in italics every time.
-paragraph 2 of section 2 results: was the diffusion performed only for the CV2 strain or for all strains? Should we expect wt to diffuse?

-uniformize strain nomenclature; e.g. CV2-DispB or CV2_DispB
- Italicize gene name in Fig 1.
- The title is different between the main text and the Supplementary Information.
- Authors list in Supp document: Change * for & for co-first authors.

- Legend Table S1 - 1st line: change "indicated" for "indicate".
- Legend Table S1- 4th line - Depleted or deleted/inactivated? According to methods, more like deleted or inactivated?

- 3rd last line of 1st paragraph results: add mpn051 in paratheses after GlpD (which should be glpD for a gene).
- 1st paragraph of results: italicize mpn133 and mpn051 in the 3rd last sentence.

- Line 3 of the 2nd section of results: Mycoplasma ovipneumoniae and arginine should be written in full because of 1st appearance in the text.

-Figure S1: The reported significant differences (WT vs CV2; p=0.07) should be reported on the figure similarly to Figure 2 (as *).

- Table S2 column 7: missing e in "Clon"?
- Legend Figure 2: Line 3: replace DS with SD.
- Legend Figure 2: Shouldn't the control be the PBS group and not the CV2 strain?
- Legend Figure 2: line 4: Correct "Data are the shown as".

- Figure 2: if the y-axis represents the gene name, italicize.

- Legend Figure S2: line 4: replace DS with SD.
- Legend Figure S2: line 1: remove space before Figure S2 title.

- Last line of Results section 3: Add a space after paragraph to separate from the next section.
- 1st line of Results section 4: "Once selected CV2 as.." syntax problem?

- First sentence of Figure S3 legend: missing punctuation ("P Server a score")?
- Line 4 Figure S3 legend: mention that this is extracellular vs intracellular ratio, otherwise we need to consult the supplemental methods to get the information.
- Last line of 1st paragraph Supp methods secretome section: "and could so"?

- Line 4 of the third paragraph of Results section 4: "allowed distinguishing proteins released in the medium (see Methods)". As this method is provided in supplementary methods, replace "see Methods" with "see supplementary Methods".

- Line 4 of the third paragraph of Results section 4: "on average by integrating the different experiments", what does that mean? Average of time points, biological replicates, or both? If you calculated an average value to identify the 90 proteins, could this value be provided in Table S3 as well? Also, highlighting the 90 significant proteins in Table S3 would facilitate the interpretation of results. Is Table S3 sorted according to something (p-value, score ?).

- Line 8 of the third paragraph of Results section 4: "we found 20 proteins that had...", do you mean "we found that of the 20 proteins that had"?

- Line 10 of the third paragraph of Results section 4: the 11 selected proteins should be highlighted in Table S3 to facilitate the interpretation of results.

- Line 13 of the third paragraph of Results section 4: "3-4 residues of these 10 proteins"; shouldn't this be 11 proteins (including the cytosolic control)? Also, I read 12 lines (excluding titles) in Table 1. Row 1-2 seems to be duplicated, resulting in a total of 11 proteins.

- Table 1: Accession names should be written as MPNXXX if proteins (like in Table S3), or mpnXXX if gene names. The column could also be called "gene identifier" instead of "accession" which required a note in the title to indicate that accession is the gene identifier.

- Last line of Table 1 legend: "5 more amino acids upstream". However, it is written at Line 13 of the

third paragraph of Results section 4 that 3-4 additional residues were included in the cloning. Which information is true?

- Line 15 and 17 of the third paragraph of Results section 4: replace "choose" with "chose". Also, replace "either we" with "we either" at line 15.

- Third paragraph of Results section 4: the fact that additional information and results about the fusion between secreted proteins and the alginate lyase is missing. Please refer to the supplementary results section.

- Figure S3: The methods describing the quantification of alginate lyase activity seem to be lacking. Also, replace commas with periods in values of the y-axis.

- Figure S3 legend: why is there an (A) in the legend? There is no panel in the figure. Also, bold the title.

- Figure S3 legend line 2: "from cultures secreting alginate lyase", I suggest replacing by "from cultures expressing alginate lyase fused to the transmembrane region of identified secreted proteins" since the alginate does not seem to be secreted in most cases.

- Supplementary results and methods: Some paragraph titles are in italic and others are not. Please make uniform.

- Supplementary results section 1: Characterization of secretion signals in different therapeutic proteins: is the text missing?

- Legend figure S3 line 6: replace "ODt 595 nm" with "OD 595nm"?

- Figure S3: Panel A: remove space between O and D for the Y-axis label. Also, in panel B, it is written CV2-DispB, while in legend it is CV2_DispB. Please correct and revise the nomenclature in the text.

- Uniformize optical density abbreviation in the text and figures.

- Figure 4 and 5: Italicise in vivo and ex vivo (as in main text). Panel A: Italicise S. aureus.
- Figure 4 title: remove « in vitro » before S. aureus.
- Figure 4 legend line 10: remove one period after catheters.
- Figure 4 legend line 12: replace "p values" with "p-values" to standardize with methods and the other legends.

- Figure 5 legend line 4: 108 CFU or 10E8 CFU? Also, italicize M. pneumoniae.
- Figure 5 C and D: Since the y-axis can represent both the increase and decrease of the SUV60 signal, change y-label for SUV60 variation or change?

- Last line of the 3rd paragraph of Results section 7: the term "promoters" seems to be missing after "endogenous". Also, Eftu is written EfTu in section 4 of the manuscript, EFTU in Table S4, and Eftu in Table 2. Please make uniform.
- 1st line of 4th paragraph of Results section 7: "Then, we checked the bacteriolytic activity of different strains on S. aureus growth curves"; where are these results? This is pretty important since you say in the next sentence that "strains with endogenous promoters showed minor effects

on S. aureus growth curve"... What about strains with P1-P5 promoters? Did they show any antimicrobial effect?

- Table 2 legend line 2: Replace Table S4D with Table S4B.
- Table 2 legend: the explanation of the method used to determine the lysostaphin concentration belongs in the methods. Regroup in the appropriate section. The method to extrapolate the date is not clearly explained. Perhaps showing the raw data with the extrapolated data would clarify things.

- Table 2: replace commas with periods in values. Replace ul by µl. Why are promoter names different from Table S4B? For example, P1 is given in Table S4B (and in the text), while P1-Lyso is provided in Table 2. Also, should we read P438 instead of pM348??

- Table S2: Why are growth curves of CV2_DispB_Lys and WT_DispB_lys reported here while these results are never mentioned in the text? Am I missing something? The information is relevant but it should be cited or put in context in the main text.

-Table S5 legend: Please correct "clonnings" and syntax.

- Next time, please add pages and/or line numbers to the manuscript.

Reviewer #3:

This manuscript describes an exciting advance in engineering therapeutic bacteria. The authors utilize recent genome editing tools they developed for the pathogen M. pneumoniae to reveal how to make it non-pathogenic, at least in a mouse model. This pathogen has benefits for therapeutic applications, namely the lack of a cell wall, thus facilitating the secretion of a wide range of therapeutic proteins. They then use a proteomic approach to discover secretion tags in this organism followed by a synthetic biology approach to repurpose those tags to secrete a protein that breaks up S. aureus biofilms. They show the efficacy of their therapeutic strain in vitro, and ex vivo and in vivo on catheters. They discover that an immune inflammatory response may help their engineered strain clear S. aureus catheter infections in vivo, an interesting new result that should motivate future technology development. This work is unique and innovative. I believe it will be widely read and inspire fruitful new directions in synthetic biology. Overall, I strongly recommend it for publication in MSB. However, there are some issues that I believe need to be resolved first (detailed below).

In the first paragraph of the results, the authors should describe the genome modification technologies used to knockout each of the four genes that they targeted. Relegating the information entirely to the supplemental compromises readability.

In the first paragraph of the results, the authors should describe how they validated that each gene was knocked out of each knockout strains.

The authors report to characterize the phenotypes of their four KO strains by quantitative proteomics and cite Table S1 as the characterization. However, Table S1 is just a list of genes, numbers, and strains presented in undefined nomenclature. The authors report data as the mean of two technical replicates, but provide no information about the variability across replicates. Furthermore, they perform no analyses of changes in protein levels or whether those changes are

statistically significant. They report in the main text that "Gene deletions were confirmed by the absence of the corresponding protein in the MS analysis". However, they do not define the units or scale they are using. What number equals absence of a protein? Given the poor presentation of Table S1 I cannot easily interpret whether or how any of the KO strains is different from the wild-type strain via proteomics. All of these issues should be corrected.

While Table S1 is a valuable resource to include with the paper, it is not a substitute for a simple figure showing the level of each target protein in the WT strain versus it's KO strain. Such a figure should be added.

There are similar issues with Table S2, which is being used to convey information about growth rate changes (or lack thereof) in their KO strains. Table S2 should be converted into a Figure to aid interpretation. All nomenclature should be defined in the legend.

Why is it significant that no CV2 colonies were found in lung homogenate after delivery to mammary tissue? Were colonies of WT M. pneumoniae found in lung homogenate after delivery to mammary tissue? Absent that control, this experiment becomes weak/superfluous.

The authors conclusion that P30 and GlpD are not major contributing factors to virulence in "mammary glands" should instead say "mouse mammary glands".

The units reported on the y-axes of Figure 2 are not defined, making the meaning of the changes in inflammatory mRNA values between WT and CT strains difficult to interpret. In the legend, the authors state "Data are the shown as mean +- DS of 2-DeltaCt*100 of at least 4 biological replicates for each sample". First, what is DS? Second, how is delta Ct calculated in this instance (what is the reference Ct value)? Why are different replicate numbers used in the different experiments? The number of replicates used should be listed for each sample.

Overall the approach taken to discover secretion tags is innovative and yields exciting results. However, there are some issues with the presentation of the method and results that need to be cleared up. First, what the di-methyl labeling approach is and the rationale for why it was chosen should be described. Second, what is meant by 24h and 72h of growth? Hours of growth in a culture media after recovery from frozen? Do these numbers correspond to a growth phase or physiological state of the organism? Third, the fact that all the information describing Figure S3, an important figure in the paper, is somewhere else (Supplementary Results Section 1) makes it rather inconvenient to interpret Figure S3. I recommend converting Figure S3 to a proper main text figure and giving it a proper legend describing what was done. Then I recommend moving the Section 1: Supplementary Results part to the methods. This will greatly increase readability.

The main text says 3-4 extra residues were added to the N-terminus of each secretion sequence, but the Table 1 legend says 5 such residues were added.

In Figure 3, what OD595 value corresponds to zero biofilm formation? A control needs to be added to indicate this value so that the effectiveness of the Dispersin B approach can be evaluated relative to a reference.

In Figure 4B-D, to what extent does the catheter stain purple/absorb at 595nm if no biofilm is ever added to it? This information is important to understand the efficacy of the Mycoplasma method and a new control should be added showing it.

The "sequences of interest" in Table S5 do not do an effective job conveying the complete sequence of the engineered plasmids, which will make it more difficult for future researchers to follow up on this work work. The complete annotated sequences of all plasmids should be uploaded to Genbank and the accession #s given.

Please find our responses (in blue) to the specific Reviewer comments in the following:

Reviewer #1:

Engineered bacteria have emerged as a promising platform for delivering therapeutic payloads in a targeted, sustained, and well-regulated manner. Criteria for selecting an appropriate bacterial chassis depends on the disease context: the engineered bacteria should be viable in the anatomical location being targeted and possess the metabolic capability to express the desired therapeutic payload. Hence, expanding our repertoire of engineerable bacteria will facilitate efforts targeting a wide array of disease.

In this study, Garrido et al. engineer an attenuated strain of Mycoplasma pnemoniae to eradicate Staphylococcus aureus biofilms. The authors knockout genes contributing to M. pnemoniae virulence in order to create an attenuated strain that is amenable to non-pathological in vivo administration. This attenuated strain is engineered to secrete Dispersin B, which demonstrates efficacy in dispersing S. aureus biofilms both in vitro and ex vivo. When the attenuated M. pnemoniae strain secreting Dispersin B does not effectively disperse S. aureus biofilms in vivo, the authors additionally include a gene for secreting Lysostaphin which consequentially improves biofilm eradication.

While the concept of using engineered bacteria to eradicate biofilm-forming pathogens is not novel (ref. 61), the study nonetheless provides advances in i) engineering a "safe" genome-reduced bacterium for therapeutic function and ii) using engineered bacteria to clear subcutaneous infections caused by biofilm-forming pathogens. However, there are a number of issues the authors should address before the work is suitable for publication:

Thanks for your overall positive feedback on the quality of the manuscript. We hope to have addressed all your concerns below. Also, we want to apologize for the extra time we needed to address the revision but the delivery of the kit to measure the adaptative immune response (Figure EV4) was delayed several times.

Major comments

1) The manuscript tends to highlight results obtained using WT and WT-DisB, which are the pathological strains. The figures and text should instead emphasize results obtained from the CV2 and CV2-DisB strains, as these are the non-pathological strains that would actually be used as a therapeutic. In particular, Fig. 3A should show a time-course for the CV2 and CV2-DisB strains (instead of WT and WT-DisB), and results section #5 paragraph #2 should reflect this change.

Thanks for this valuable suggestion. We agree with the Reviewer that the results obtained with the non-pathological strain (CV2) should be emphasized. However, as the performance of WT and CV2 was similar for all the experiments, except for those carried out *in vivo,* we feel that it

is important to keep the data of WT experiments in all the figures, to highlight the contrast between *in vivo* and *in vitro* or *ex vivo* experiments. Thus, in the text, we now emphasize the results obtained with CV2 strain, and add a comment at the end of each section mentioning that similar results were obtained with WT. Similarly, in all figures, the first panels are dedicated to the results obtained with CV2 strain, and subsequent panels detail those of WT strain. We believe that the current version of the manuscript represents a good trade-off between highlighting the results of the CV2 strain and maintaining those of WT to reflect how things could change between *in vivo* and *in vitro* experiments. Specifically, the requested time-course of CV2 and CV2-DispB strain is shown as Figure 4A, and the same time-course based on WT and WT-DispB, as Figure 4B.

2) For in vivo studies, the authors use PET scans to track S. aureus biofilm production. While this technique allows comparisons between different treatment groups and facilitates longitudinal examination, it does not provide specific information about the absolute change in S. aureus abundance or biofilm production. Instead, this PET signal is seemingly dependent on a number of factors including bacterial abundance and inflammation (ref. 43). Ideally the authors would also provide CFU counts to demonstrate absolute changes in S. aureus abundance or discuss how PET signal corresponds to absolute S. aureus abundance. For example, what does a 20% increase or decrease in PET signal correspond to in terms of S. aureus abundance?

We agree with the referee that PET results provide a multifactorial signal influenced not only by *S. aureus* abundance but also by inflammation, immune cell infiltration, etc. Accordingly, we added a sentence in the manuscript pointing to what is actually measured by the PET signal (lines 394-395). In any case, for the specific question raised by the referee, the answer can be extracted from the original paper in which *S. aureus* biofilms were tracked by PET (Garrido *et al* 2014). Mice treated with rifampicin showed a decrease in SUV60 signal of around 30% (from 1.66 to 1.15) at day 7 of the treatment; this corresponds to a decrease of around 2.5 orders of magnitude in the CFUs of *S. aureus*. Hence, a decrease of 20% in the SUV signal could be taken as a decrease of two orders of magnitude in *S. aureus* living cells. We included a sentence in the results highlighting this data (lines 401-402), and we have added here the figure of the cited paper with these results to facilitate the reviewing process:

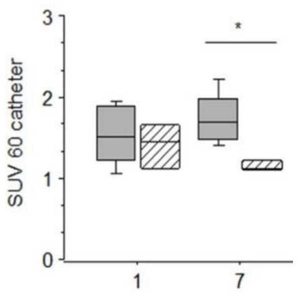

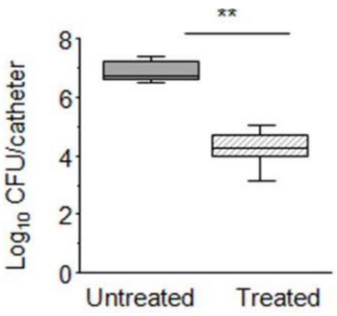

Figure 5A of the article PMID: 25155589 (https://pubmed.ncbi.nlm.nih.gov/25155589/): "Evaluation of rifampin treatment effect using [^{18}F]FDG-MicroPET imaging in mice carrying sealed catheters precolonized with S. aureus strain ATCC 15981

3) The authors claim that the

attenuated CV2 strain is non-pathological; however, it seemingly still elicits an immune response (Fig. S1). The authors should address whether the fact that the CV2 strain still elicits a weak immune response precludes it from long-term or repeated use. For example, is there evidence that no adaptive immune response will be mounted against the attenuated strain?

This is a very interesting question. To address this, we could not use the mammary gland model, in which the immune response and virulence were measured, since for this, female mice need to be lactant, or else the glands involute. For short-term experiments, such as the one of virulence assessment, the offspring are sacrificed, but at four days post-infection, the mammary gland is still perfectly formed. For a long-term experiment involving repetitive dosage, we should maintain the offspring alive to avoid mammary gland involution, but in this scenario we cannot assure that *Mycoplasma* is not removed from the injection site due to lactation of the offspring, which could mask the results of a possible immune response. To avoid this, we decided to carry out this experiment by doing either one or two subcutaneous injections and measured local inflammation at the injection point, IL-4, IFN-g and the generation of IgG and IGM antibodies against the WT or the CV2 strain. We have added a new supplementary figure (Figure EV4) with the results of such an experiment. Essentially, we see an inflammatory response at the point of injection with the WT strain, but not with the CV2 strain, in agreement with what we see with the mammary glands. We don't see an increase of IL-4 and IFN-g with either of the strains, and there is no significant production of IgM against them. However, we see a significant IgG response against both strains following a second injection. This could indicate that repeated injections of CV2 won't trigger inflammation, but they do result in IgG production, which could affect its efficacy. We have now added a paragraph in Results where we present these results (lines 267-285) and mention it in the Discussion (lines 495-507).

4) The final demonstration (Fig. 5D) using the CV2-DisB-Lyso strain performs inconsistently in vivo (4/11 replicates showed no decrease in S. aureus loads). A clear explanation of how this is an improvement over existing methods therapies is warranted. Additionally, the authors should discuss improvements that would facilitate the use of CV2-DisB-Lys as a more robust therapy.

Several reasons could explain the inconsistency in the outcome of treatments based on CV2-DispB-Lys cells. One is inherent to the model of the subcutaneous catheters, as it depends on the behaviour of the particular mouse; here, the catheter might move away from the place in which mycoplasma was administered, hindering the therapeutic effect. Indeed, the results obtained with the WT-DispB strain are more consistent between different animals, which might suggest that inflammation created by WT strain limits the movement of the catheter. Of note, this subcutaneous setting is not the natural niche of M. pneumoniae, which probably limits its capacity to spread within the tissue and to compensate for the possible movement of the catheter. Therapies based on systemic administration of recombinant proteins might not face this limitation but are certainly more prone to adverse effects. An alternative explanation for this inconsistency is the possible appearance of resistance to lysostaphin, a phenomenon that is quite common in S. aureus (Gründling et al, 2006). Hence, a possible way to improve a therapy based on the CV2 strain would to combine dispersin and lysostaphin with other

antibacterial peptides (e.g. CHAPK, LysK), thereby overcoming any possible appearance of resistance by overwhelming the adaptation capacity of pathogenic bacteria.

We now mention these points in the Discussion section (lines 571-586).
Minor comments

1) The authors offer no definition or explanation of the standardized uptake value (SUV), except for a mathematical equation in the supplement. The results reported in Fig. 5 would be more accessible to a broader audience if the authors qualitatively defined the SUV60 in the main text and described what the SUV measures (i.e. bacterial abundance and inflammation).
We apologize for this; we now define SUV60 in the text (lines 394-397):

To obtain a qualitative estimate of the bacterial abundance, immune cell infiltration and inflammation, we determined the SUV60 signal (see Methods). The efficacy of the treatment was expressed as the percentage of variation of the SUV60 signal obtained in the catheter area from 1 to 4 days after treatment.

2) The authors should comment on biocontainment characteristics for M. pneumoniae. For example, how long is *M. pneumoniae* expected to colonize? Ideally *M. pneumoniae* will only be present throughout the therapeutic course and will not persist in the body.

This is an interesting question. Although we don't have data of the persistence of *M. pneumoniae* in a subcutaneous setting, we know that upon intratracheal administration, the WT strain completely disappears from lung tissue after 4 days, and does not spread to other organs. However, we believe that providing these data would not add to the manuscript, as the lung is out of the scope of our present work. Of note, in humans, *M. pneumoniae* is only capable of causing disease in infants, elderly, and immunocompromised people, so most likely its persistence in subcutaneous settings (which is not their natural niche) must be quite limited.

Regarding the biocontainment characteristics, we added a paragraph to the Discussion section (lines 590-600) on possible strategies to limit its growth on demand, such as the use of almost any non-beta-lactam antibiotics, or the inclusion of an inducible promoter to control the expression of key components to the cell (such as the cell division operon or the RNApol operon). We foresee that any of these options would be easy and effective.

3) The authors allude to the fact that S. aureus growth curves are used to assess efficacy of Lysostaphin-killing. However, these growth curves do not appear to be in the main text or supplementary files. These growth curves should be included to demonstrate the effect CV2-DisB-Lys has on S. aureus growth.

We apologize for this; this figure can be found in the current submission as a supplementary figure termed Figure EV5.

4) In addition to treating biofilms with systemically administered biomolecules, there have been growing advances in using phage to treat biofilms (Khalifa et al. (2015) Applied and Environmental Microbiology; Lu et al. (2007) PNAS). The authors should acknowledge some of these efforts and discuss the advantages of using bacterial therapies.

Thanks for this valuable suggestion; we have now included these references in the Discussion section and briefly commented on the advantages of bacterial therapies over phage therapies (lines 586-589)

5) The authors claim, "No significant changes were found in the doubling times of the generated strains ..." but do not provides statistical analyses to validate their use of "significant". Similarly, the authors state: "... the cytotoxic nuclease (mpn133) and CARDS toxin (mpn372) contribute to M. pneumoniae virulence; and have a synergistic effect" yet do not provide any quantitative analyses to validate their use of "synergistic". The authors should either provide quantitative support for this terminology or replace these terms.

Regarding the doubling times, we decided to repeat this experiment and calculate all of them (and not take data from published articles for some of the strains). We did three independent biological replicas (lines 191-193):

Similar doubling rates were observed (~9 h) for all the strains except for those carrying deletions in mpn133 gene, in which the doubling rates were slightly but significantly increased (~10 h), as compared to the wild-type (WT) strain.

Regarding the term "synergistic", it would have been more accurate to say "additive"; in any case, we rewrote the paragraph and avoided the use of this terminology to avoid misinterpretations (lines 220-223).

6) At the end of the introduction, the authors introduce the term "Mycochassis" to denote their attenuated strain. However, this term is never used again. We recommend removing the term "Mycochassis".

Thanks for pointing this out; we have removed it.

7) The authors claim, "data for mpn133 and mpn051 mutants was obtained from previous work" - this previous work needs to be cited.

As stated above (response to question 5), in order to properly compare the growth of all the strains, we now avoid taking data from previous papers and have assessed the growth of all the strains in parallel under the exact same conditions. Consequently, we removed this sentence.

8) The order of conditions in Fig. 1C should be the same as in Fig. 1A. Currently, the Δ*mpn372* and delta mpn133 are not in the same order.

We apologize; we have now changed the order of the strains to be consistent in all the panels.

9) The authors should remove "beautifully" in the following sentence to help omit flowery and unnecessary language: "This is beautifully exemplified in this work by the comparison between cell and supernatant treatments."

We agree and have removed it.

10) There is a typo in the methods: "A detailed list of plasmids and the sequence of the different modules included on them is available on Table S6." Table S6 contains the qPCR primers and not the plasmid sequences.

Thanks for pointing this out; we apologize for the mistake. In the current submission, a detailed list of the primers used to generate the ssDNA recombineering substrates, and the plasmids generated in this work (including the sequence of the different modules included on them), is available in Dataset EV6. Dataset EV7 contains information of the primers employed for qPCR.

11) The scale bar in Fig. 5 should have a title (e.g., "SUV60).

Apologies; we have now modified the figure to add the title.

12) The authors should provide sequences for the 5'UTR and 3'UTR regions used to express proteins, such that the work can be reproduced or adapted.

It should be noted that *M. pneumoniae* does not require RBS sequences to translate properly mRNA and, in consequence, 5'UTR regions are either absent or really short. In any case, we believe that the information provided in Dataset EV6 in the current version provides extensive information about the sequences of the most important plasmids of the study. In addition, these sequences have been uploaded to ENA, and the reference numbers are provided in the Data Availability section. Lastly, for other less-relevant plasmids (e.g. those assembled to screen the activity of different secretion signals), extensive information of their sequence is provided in Dataset EV4

13) There are a couple of sentences that are difficult to understand and need to be reworded. I've listed them below:

- "Moreover, the absence of cell wall also facilitates direct release of secreted bioactive compounds, which might be combined when required with antibiotics targeting cell wall formation without affecting the viability of the therapeutic Mycoplasma strain."

We rewrote the sentence as follows (lines 118-123):

The lack of a cell wall is important for several reasons: it is likely to lessen detection by the host immune system, as cell wall components are generally major targets of immune system recognition (Sukhithasri et al, 2013); it facilitates the direct release of secreted bioactive compounds; and it provides the possibility of targeting the cell wall of a pathogenic bacteria by using a combination of antibiotics together with a mycoplasma-based therapeutic vector.

- "As up to 90% of the total biofilm thickness can be attributed to this matrix, S. aureus cells encounter in these structures a great protection against antimicrobial agents and host defence mechanisms."

We have replaced this with (lines 148-149):

S. aureus cells within these structures are strongly protected against antimicrobial agents as well as the host defense mechanisms (del Pozo & Patel, 2007)

Reviewer #2:

Garrido et colleagues report the creation of attenuated Mycoplasma pneumoniae strains as live biotherapeutic chassis to treat bacterial infections. They first show that deleting selected genes encoding virulence factors results in colonization of the mouse mammary gland without

causing macroscopic lesions. The authors also investigate the production of cytokines by the mammary gland tissue by RT-qPCR. The next step was to perform mass spectrometry and bioinformatics analyses to identify secreted proteins along with their respective signal peptides. Using the mpn142 signal peptide, dispersine B was introduced in wild-type or attenuated (CV2) M. pneumoniae chasses, and their ability to disrupt biofilms was assayed in vitro and ex vivo. Finally, the authors performed an in vivo assay using a mouse model of catheter infection. The results indicate that the attenuated chassis was not as efficient as the wild-type in this context, leading to an experiment where both the WT and CV2 strains were combined. A CV2 strain producing dispersine B and lysostaphin also generated better results than CV2 secreting dispersine B alone.

Globally, I found the rationale of the project interesting and I believe there is an expanding readership for this type of work To my knowledge, there is no other group engineering mycoplasma species as living medicines. Using a near-minimal well less bacterium as a live biotherapeutics could indeed provide a new approach to fight bacterial pathogens. However, many aspects are difficult to understand and there is an important "lack of finish" that decreases the overall quality of the manuscript. There are many typos, errors, sentence the are missing a few words or details that are not clearly explained. The manuscript thus appears to lack important information needed to appreciate the data and the overall work. The authors should also be careful about the wording of some claims. I am detailing some of these issues below.

Thanks for your overall positive feedback on the quality of the manuscript. We hope to have addressed all your concerns below. Also, we want to apologize for the extra time we needed to address the revision but the delivery of the kit to measure the adaptative immune response (Figure EV4) was delayed several times.

---MAJOR COMMENTS---

-Rewording would be required to tone down certain claims in the title and abstract:

o The title can be misleading. M. pneumoniae has a naturally small genome but the chassis presented here is not minimal per see.

o Abstract: M. pneumoniae does recombine. There is evidence of recombination in the literature (e.g. PMID: 20543037). The introduction offers more nuance about this but the abstract is misleading.

o Abstract: The genetic code does not prevent potential genetic exchanges between mycoplasma.

Thanks for your suggestions. Regarding the abstract, we agree with you that it could be misleading so we replaced the term minimal for "genome-reduced". Regarding the recombination issue, we decided to remove it from the abstract and leave it just in the Introduction section. However, we would like to highlight that the strain used here is M129-B7; in the suggested reference, it has an almost negligible recombination capacity (a single clone obtained). In any case, in the Introduction section (where we comment on the limited

recombination capacities of this bacterium), we toned down the sentence by changing "blocking" to "limiting", and introduced the suggested reference (lines 116-117). In this way, the reader can see that, despite the lack of a functional Holliday's junction resolvase (Sluijter *et al* 2010; Sluijter *et al* 2012), a barely-detectable recombination activity can still be observed in this strain (Krishnakumar et al, 2010), as suggested). Finally, we also rewrote the abstract to highlight that its genetic code prevents horizontal gene transfer to most bacterial genera (rather than to other bacteria, as previously stated) (lines 50-51).

-Several genes were deleted to obtain an attenuated strain of M. pneumoniae. The phenotypes of the resulting strains were analyzed by mass spectrometry. Why not sequence the genomes of the strains to confirm these deletion events? Was this done? The wording suggests that genotyping was not performed. Mass spectrometry alone could be misleading as it is possible that there would be no expression under those laboratory conditions.

In the revised version, a PCR screen of the different deletions performed can be found in Figure EV1B. In addition, we sequenced the CV2 genome (which is actually the most important strain of the article) and that of WT as a reference. Sequencing data are available from a public database (European Nucleotide Archive), as indicated in the Data Availability section.

- The authors state that there were no significant changes in the doubling times of the various mutant strains. How can we be sure about this as there is no growth curve presented and there are no standard deviations presented with the data? There is almost a two-hour difference between the slowest- and the fastest-growing mutant strains.

We apologize for this. For this resubmission, we determined the growth of all the mutant strains used in this work in parallel (rather than using data from previous published work, as before) by measuring the increase in protein content over time, with three biological replicas. We observed a slight yet significant increase in the doubling times of those strains carrying a deletion in the *mpn133* gene (i.e. Δ*mpn133* and CV2 strains). These results can be found in Figure EV1D and Dataset EV2. We appreciate your feedback on this issue, as now we are quite confident the results are accurate. We also rewrote the paragraph accordingly (lines 189-193)

-The immune response of the mammary gland tissue was investigated by qPCR. Although other assays may arguably offer a better assessment of the effective response, I understand that RT-qPCR is a more accessible approach to answer this question. However, I doubt that the comparative threshold cycle (Ct) method was applied correctly. This method generally uses the is usually 2 (or whatever the reaction efficiency is) to the power of the delta-delta Ct, and the result should be a fold change. The equation presented here seems to include only a delta Ct and is multiplied by 100, which results in some cases in very low numbers (between 0.05 and 0.5 with large error bars in the case of IL-6). There is no reference for the method that was applied. This should be clarified or calculated with a more standard approach.

Our apologies; in the previous version, the Ct method was applied using the following formula:

$$2^{-\Delta Ct} \text{ x } 100 = [2^{-(\text{Mean Ct gene unknown} - \text{Mean Ct gene housekeeping})}] \text{ x } 100$$

In other words, the values were not normalized to the ones obtained in the PBS control group

(and therefore, the PBS group was not always 1, and the values seemed low). In any case, the Reviewer is correct in stating that the delta-delta CT method is more standard and allows an easier comparison between groups. Thus, in the revised version, we used the Ct method as follows:

$$\Delta Ct = \text{Mean Ct analyzed gene - Mean Ct } gapdH$$

$$\Delta\Delta Ct = \Delta Ct \text{ treated group - } \Delta Ct \text{ PBS group}$$

$$\text{Relative abundance} = 2^{-\Delta\Delta Ct}$$

We have changed the corresponding figures accordingly.

-The section about the identification of the secretion signals (section number 4) is very difficult to follow, in part because Table S3 is not clearly presented. How is the table sorted and why (see below for additional points on supplementary data)? It is thus hard to understand what proteins the authors refer to when mentioning:11 proteins of which 6 were in the top 20 and 4 were predicted to be membrane proteins. The authors also indicate in the text that 3-4 residues were added in addition to the predicted signal peptides but Table 1 rather mentions 5 residues (and these residues are not clearly indicated in Table 1). The strategy and results should be easier to follow.

We apologize that this information was difficult to follow. We have now reorganized Table S3 (now, DataSet EV3) to make it easier to interpret. Dataset EV3 is sorted by the summatory of extracellular/intracellular MS ratios of the 4 datasets analyzed. The proteins selected to test the capacity to secrete heterologous proteins when fused to their N-terminal sequences are highlighted in blue. Regarding the number of extra residues added after the cleavage site, there was a mistake in the previous version, and we apologize for it. We checked the sequencing results of these constructs and can verify that all of them have 6 extra residues after the predicted cleavage site. The exact sequences that were tested are now shown in Dataset EV4.

-It looks like some data is missing from the manuscript about S. aureus growth curves (1st line of 4th paragraph of Results section 7): "Then, we checked the bacteriolytic activity of different strains on S. aureus growth curves"; Where are these results? This is pretty important since the next sentence indicates that "strains with endogenous promoters showed minor effects on S. aureus growth curve"... What about strains with P1-P5 promoters? Did they show any antimicrobial effect?

We apologize; this growth curve is now shown in Figure EV5. In general, synthetic promoters outperformed naturally-existing ones, with P3 the one that produced the highest levels of lysostaphin.

-The supplementary information is not clearly presented.

-Table S1:

o No context to understand the information presented in the first line

We agree; we have now explained in the legend that the first line is the reference to the raw mass spectroscopy data uploaded in the PRIDE database.

o What is the difference between an empty cell and a value of zero?

We apologize: there was no difference. In any case, we decided to change the format of the table, so that it contains neither empty cells nor zeros but rather ND (not detected) for cases in which a protein was not detected in either of the two technical replicates. In addition we added a column for each strain showing the Standard deviation in the AUC values obtained in the two technical replicates. Consequently, cases in which the standard deviation could not been calculated given the absence of protein detection in either of the replicates are now indicated by NC (non-calculated).

o What are the last three columns?

The last four columns were the MS results of both the cells and supernatants of the WTDispB-Lys and CV2DispB-Lys strains. For the sake of clarity, we have now removed these data, given that none of these strains have been introduced at the point in the text where the table is cited.

o Why use the area under the curve for the 3 most abundant peptides instead of normalized spectral abundance?

Using the areas of the three most abundant peptides is a robust and precise procedure to estimate protein abundances in proteomics studies; for instance, it was used in JC Silva et al, in MCP 2006, in which they demonstrated its superiority to normalized spectral abundances (e.g. EmPAI) in a wider range of protein concentration. (https://doi.org/10.1074/mcp.M500230-MCP200)

-Table S2:

o No growth curve is shown in the manuscript; these are growth parameters.

We believe that 430/560 growth curves are more suitable to measure metabolic activity than actual growth. Therefore, we are more confident with the determination of growth parameters based on protein content increase over time. We corrected the manuscript and removed the term "growth curve", as you are correct that it is not shown anywhere. We now show the growth parameters of all the strains (obtained in parallel, with three independent cultures per strain) in Dataset EV2, and a graphic representation of the doubling times in Figure EV1D.

o How qPCR measurements described in the methods were converted into biomass?

We measured biomass independently of the qPCR measurements at the beginning and end of the experiment. In any case, we realized that automated extraction of gDNA for the qPCR added a lot of noise to the results, so we decided to just use data of the protein content increase. The formulas to calculate the doubling times are indicated in the Methods.

o How can we know if there is a significant difference in the doubling time if there is no standard deviation?

This is now corrected; dataset EV2 now shows the doubling times of each strain with standard deviation calculated from three independent cultures per strain.

-Table S3:

o How is this table sorted? This is extremely difficult to understand with the current explanations.

Apologies; as stated above, we now order the genes by the summatory of the ratios (supernatant/pellet) to make the analysis clearer. The genes for which the 5' end was fused to the coding sequence of alginate lyase are labelled in blue.

o There are two T1-2 columns for the p-value

Sorry, it was a typo. In addition, we now changed the table to refer to the times points as T24 and T72h, rather than T1 and T2.

o What is the difference between an empty cell and a value of zero?

Apologies for being unclear. In the current version, we now denote ratios that could not be determined due to the absence of protein detection in either the supernatant or the cell pellet as NA (not available). Similarly in the columns of p-value, NC (non-calculated) is shown for cases in which the p-value could not be determined due to the absence of protein detection in one of the fractions. Finally, zeros are shown for p-values less than 0.000001, as stated in the legend.

-Figure S1: how is the score defined? Was this performed in a double-blind manner? This type of subjective data should be blinded.

We apologize for not being clearer at this point. Yes, the histopathological score was determined in a blind manner—e.g., the team that performed the analysis (Veterinary Faculty of Zaragoza University, Spain) did not know the identity of the samples. This is now indicated in Methods (lines 721-724)

o How can we know if there is a significant difference in the doubling time if there is no standard deviation?

Apologies once again; as stated above, this is now corrected. Dataset EV2 now shows the doubling times of each strain with standard deviation calculated, from three independent cultures per strain. This dataset also shows the statistical significance in the doubling times calculated for each strain.

-Figure S3:

o there is no title on the x-axis. One can deduce that these are gene numbers for the signal peptide but this should be explicit in the figure or the figure legend. Indeed, you are right that the numbers indicated the gene numbers for the signal peptide. In any case, we now indicate this clearly in the figure as well as in the figure legend. Note that this figure is now part of the main figures of the manuscript, as requested by one of the reviewers.

---MINOR COMMENTS---

There are several typos, missing words, etc. throughout the documents that make the work more difficult to appreciate.

We have now sent it to a native English speaker for corrections.

-Figure 1A: give the +++ / - lesion score in A instead of C ? Why are the gene deletions not presented in the same order between panel A and panel C? Also, why give the p-value thresholds in legend if no difference was observed?

Thanks for pointing this out. It is true that it makes more sense to show the lesion score with the pictures and not with the CFU counts. The order of the strains in the different panels has also been fixed. Finally, we removed the thresholds for the p-values from the legend, as it is true that showing them here was not useful at all.

- Figure 2 and Legend Figure 2: 2-ΔCt*100 (legend) and 2ΔCtx100 (figure)? I do not understand these units. Normally, we provide RT-qPCR results as relative expression values (fold change) using the 2^-ΔΔCt formula, and arbitrarily setting the control group to a value of 1 (PBS). Also correct Figure S2 and methods accordingly.

Apologies, as stated above we agreed with you that the 2^-ΔΔCt formula, is a more standard way to represent the data so we did it like that.

- Mass spectrometry: why use area under the curve for the 3 most abundant peptides instead of peptide count / of NSAF?

The MS facility of our institute always uses the AUC for the 3 most abundant peptides as a measure. We inquired about your concern and they were convinced about the superior accuracy of this method compared to normalized spectral abundances. A reference for this can be found here: (https://www.ncbi.nlm.nih.gov/pmc/articles/PMC3775642/)

- The results section describing the construction of transmembrane proteins fused to the alginate lyase protein (section 4) is very difficult to understand clearly. Please revise the text as many errors and syntax problems are present. A figure summarizing the design would also greatly help the comprehension of this section.

Apologies we have written again this section (lines 298-323). We do not see clearly which type of scheme could help the comprehension of the section. What we did is to move Figure S3 to be part of the main figures (Figure 3), and clarify into the table Dataset EV4 the exact sequences employed to fuse to alginate lyase. We hope that this, together with the rewriting of the alginate lyase section in the main text is enough to increase the comprehension of the paragraph.

- italicize in vivo in title and abstract as for the rest of the paper. Gene names should also be in italics every time.

Apologies. Done.

-paragraph 2 of section 2 results: was the diffusion performed only for the CV2 strain or for all strains? Should we expect wt to diffuse?

Sorry, it was done for CV2 and WT now it is specified in the text (lines 216-219):

We then evaluated the diffusion of the WT or CV2 strains from the mammary gland to the lung, kidney, spleen, liver or blood, by spreading a homogenate of these tissues on agar plates. No colonies were found, confirming that the mycoplasma cells did not diffuse away from the site of delivery.

-uniformize strain nomenclature; e.g. CV2-DispB or CV2_DispB

- Italicize gene name in Fig 1

Done.

- The title is different between the main text and the Supplementary Information.

Apologies, anyway, following the journal instructions there is not anymore supplementary results.

- Authors list in Supp document: Change * for & for co-first authors.

Apologies, anyway, following the journal instructions there is not anymore supplementary results.

- Legend Table S1 - 1st line: change "indicated" for "indicate".

Corrected.

- Legend Table S1- 4th line - Depleted or deleted/inactivated? According to methods, more like deleted or inactivated?

Deleted. Sorry for that.

- 3rd last line of 1st paragraph results: add mpn051 in parentheses after GlpD (which should be glpD for a gene).

Solved.

- 1st paragraph of results: italicize mpn133 and mpn051 in the 3rd last sentence.

Done.

- Line 3 of the 2nd section of results: *Mycoplasma ovipneumoniae* and arginine should be written in full because of 1st appearance in the text.

Totally true! Thanks for pointing it out.

-Figure S1: The reported significant differences (WT vs CV2; p=0.07) should be reported on the figure similarly to Figure 2 (as *).

We have not added the * symbol since the value for P (0.07) is higher than the cutoff value of 0.05 we used in the paper. In any case we have added a horizontal Bar and put the p value of 0.07 between the two samples analysed.

- Table S2 column 7: missing e in "Clon"?

We changed this table and this information that was not relevant has been removed.

- Legend Figure 2: Line 3: replace DS with SD.

Done.

- Legend Figure 2: Shouldn't the control be the PBS group and not the CV2 strain?

Absolutely. That was a mistake. Thanks for spotting it.

- Legend Figure 2: line 4: Correct "Data are the shown as".

Rewritten.

- Figure 2: if the y-axis represents the gene name, italicize.

Done.

- Legend Figure S2: line 4: replace DS with SD.

Done.

- Legend Figure S2: line 1: remove space before Figure S2 title.

Done.

- Last line of Results section 3: Add a space after paragraph to separate from the next section.

Done.

- 1st line of Results section 4: "Once selected CV2 as.." syntax problem?

Rewritten.

- First sentence of Figure S3 legend: missing punctuation ("P Server a score")?

Rewritten.

- Line 4 Figure S3 legend: mention that this is extracellular vs intracellular ratio, otherwise we need to consult the supplemental methods to get the information.

Rewritten.

- Last line of 1st paragraph Supp methods secretome section: "and could so"?

Rewritten.

- Line 4 of the third paragraph of Results section 4: "allowed distinguishing proteins released in the medium (see Methods)". As this method is provided in supplementary methods, replace "see Methods" with "see supplementary Methods".

We have written this whole section again. Note that no supplementary methods are allowed in this journal so we moved everything to methods.

- Line 4 of the third paragraph of Results section 4: "on average by integrating the different experiments", what does that mean? Average of time points, biological replicates, or both? If you calculated an average value to identify the 90 proteins, could this value be provided in Table S3 as well? Also, highlighting the 90 significant proteins in Table S3 would facilitate the interpretation of results. Is Table S3 sorted according to something (p-value, score ?).

We have written again this whole section and we think now is more clear. Basically we selected some proteins to test their capacity to secrete heterologous proteins based on the degree of enrichment on the culture supernatants, the reproducibility of this enrichment across the four data sets, and their *in silico* predictions to be secreted, Regarding Table S3 we

reorganized it. Now it is sorted by the summatory of the Out/In MS ratio and the proteins showing a value in this summatory higher than 13 (i.e., 22 in total) are highlighted in yellow in the location column.

- Line 8 of the third paragraph of Results section 4: "we found 20 proteins that had...", do you mean "we found that of the 20 proteins that had"?

We have written this whole section again.

- Line 10 of the third paragraph of Results section 4: the 11 selected proteins should be highlighted in Table S3 to facilitate the interpretation of results.

Done (in blue in the gene identifier column as indicated in the legend).

- Line 13 of the third paragraph of Results section 4: "3-4 residues of these 10 proteins"; shouldn't this be 11 proteins (including the cytosolic control)? Also, I read 12 lines (excluding titles) in Table 1. Row 1-2 seems to be duplicated, resulting in a total of 11 proteins.

Sorry for the mistake. This is now solved. As stated in the text and showed in the tables the number of signals tested were 9, plus the cytosolic control.

- Table 1: Accession names should be written as MPNXXX if proteins (like in Table S3), or mpnXXX if gene names. The column could also be called "gene identifier" instead of "accession" which required a note in the title to indicate that accession is the gene identifier.

Done.

- Last line of Table 1 legend: "5 more amino acids upstream". However, it is written at Line 13 of the third paragraph of Results section 4 that 3-4 additional residues were included in the cloning. Which information is true?

Apologies and thanks for making us double check this. As stated before we check the sequencing results of all the fusions to A1-III and confirmed that the number of extra residues added after the predicted cleavage site is 6. The exact sequences can be found in Dataset EV4.

- Line 15 and 17 of the third paragraph of Results section 4: replace "choose" with "chose". Also, replace "either we" with "we either" at line 15.

Rewritten.

- Third paragraph of Results section 4: the fact that additional information and results about the fusion between secreted proteins and the alginate lyase is missing. Please refer to the supplementary results section.

According to the format of the journal no supplementary results section is allowed. We included all the information in the main text and extended the corresponding datasets and methods. Specifically, Dataset EV4 contains all the information regarding the constructs of alginate lyase fusions in a detailed manner. Also, a new paragraph in methods has been included to explain the screening of the different secretion signals by means of alginate lyase activity (lines 838-846).

- Figure S3: The methods describing the quantification of alginate lyase activity seem to be lacking. Also, replace commas with periods in values of the y-axis.

This Figure is now Main Figure 3 as requested by one of the reviewers and the comma/period issue was solved. We included a specific section in methods describing the quantification of alginate lyase activity (lines 838-846).

- Figure S3 legend: why is there an (A) in the legend? There is no panel in the figure. Also, bold the title.

Moved to Main Figure 3 and solved.

- Figure S3 legend line 2: "from cultures secreting alginate lyase", I suggest replacing by "from cultures expressing alginate lyase fused to the transmembrane region of identified secreted proteins" since the alginate does not seem to be secreted in most cases.

We have changed the legend as follows:

Figure 3*. Validation of secretion signals with alginate lyase A1-III (A) Plot showing results of the turbidimetric assay (OD 300 nm) conducted to evaluate alginate lyase activity present in the culture supernatants (SN) of the indicated strains carrying the alginate lyase A1-III coding sequence fused to different secretion signals. Culture supernatants (n=1) were collected at 0 h, 24 h or 48 h post-inoculation as indicated. (B) Comparison of the turbidimetric assay results obtained with mpn142 and mpn142Opt secretion signals. Culture supernatants (n=1) were collected at 0 h, 5 h or 24 h post-inoculation as indicated. WT strain and medium (Hayflick) were added as controls.*

- Supplementary results and methods: Some paragraph titles are in italic and others are not. Please make uniform.

To fit into the journal style there are no supplementary results or methods anymore. We included all the information into the main text taking care of the format.

- Supplementary results section 1: Characterization of secretion signals in different therapeutic proteins: is the text missing?

This part is now included in the main text.

- Legend figure S3 line 6: replace "ODt 595 nm" with "OD 595nm"?

Apologies for the mistake. Corrected.

- Figure S3: Panel A: remove space between O and D for the Y-axis label. Also, in panel B, it is written CV2-DispB, while in legend it is CV2_DispB. Please correct and revise the nomenclature in the text.

We have corrected the legend and the figure.

- Uniformize optical density abbreviation in the text and figures.

Thanks for pointing out. Solved.

- Figure 4 and 5: Italicise in vivo and ex vivo (as in main text). Panel A: Italicise S. aureus.

Done.

- Figure 4 title: remove « in vitro » before S. aureus.

Rewritten.

- Figure 4 legend line 10: remove one period after catheters.

Done.

- Figure 4 legend line 12: replace "p values" with "p-values" to standardize with methods and the other legends.

Done.

- Figure 5 legend line 4: 108 CFU or 10E8 CFU? Also, italicize M. pneumoniae.

Corrected.

- Figure 5 C and D: Since the y-axis can represent both the increase and decrease of the SUV60 signal, change y-label for SUV60 variation or change?

Thanks for pointing out. Changed.

- Last line of the 3rd paragraph of Results section 7: the term "promoters" seems to be missing after "endogenous". Also, Eftu is written EfTu in section 4 of the manuscript, EFTU in Table S4, and Eftu in Table 2. Please make uniform.

All uniformized to *mpn665* gene.

- 1st line of 4th paragraph of Results section 7: "Then, we checked the bacteriolytic activity of different strains on S. aureus growth curves"; where are these results? This is pretty important since you say in the next sentence that "strains with endogenous promoters showed minor effects on S. aureus growth curve"... What about strains with P1-P5 promoters? Did they show any antimicrobial effect?

Apologies. This graph can be found in Figure EV5.

- Table 2 legend line 2: Replace Table S4D with Table S4B.

Corrected.

- Table 2 legend: the explanation of the method used to determine the lysostaphin concentration belongs in the methods. Regroup in the appropriate section. The method to extrapolate the date is not clearly explained. Perhaps showing the raw data with the extrapolated data would clarify things.

As this issue was controversial and the manuscript was already quite extensive after moving supplementary methods and results to the main text in compliance to the journal format, we decided to remove these calculations of Lysostaphin concentration. Indeed, we believe that just by taking a look at the Figure EV5 the reader could get an estimation of the amount produced in a quick view.

- Table 2: replace commas with periods in values. Replace ul by µl. Why are promoter names different from Table S4B? For example, P1 is given in Table S4B (and in the text), while P1-Lyso is provided in Table 2. Also, should we read P438 instead of pM348??

Corrected.

- Table S2: Why are growth curves of CV2_DispB_Lys and WT_DispB_lys reported here while these results are never mentioned in the text? Am I missing something? The information is relevant but it should be cited or put in context in the main text.

Sorry about that. As you mentioned when Dataset EV2 is mentioned in the text only the different mutant strains (Δ*mpn051*, Δ*mpn133*, Δ*mpn372* Δ*mpn453* and CV2) have been introduced to the reader. As we had to repeat the growth measurements by triplicates of all these strains we decided to circumscribe the information presented in Dataset EV2 to these strains.

-Table S5 legend: Please correct "clonnings" and syntax.

Done

- Next time, please add pages and/or line numbers to the manuscript.

Apologies, it won't happen again!

Reviewer #3:

This manuscript describes an exciting advance in engineering therapeutic bacteria. The authors utilize recent genome editing tools they developed for the pathogen M. pneumoniae to reveal how to make it non-pathogenic, at least in a mouse model. This pathogen has benefits for therapeutic applications, namely the lack of a cell wall, thus facilitating the secretion of a wide range of therapeutic proteins. They then use a proteomic approach to discover secretion tags in this organism followed by a synthetic biology approach to repurpose those tags to secrete a protein that breaks up S. aureus biofilms. They show the efficacy of their therapeutic strain in vitro, and ex vivo and in vivo on catheters. They discover that an immune inflammatory response may help their engineered strain clear S. aureus catheter infections in vivo, an interesting new result that should motivate future technology development. This work is unique and innovative. I believe it will be widely read and inspire fruitful new directions in synthetic biology. Overall, I strongly recommend it for publication in MSB. However, there are some issues that I believe need to be resolved first (detailed below).

Thanks for your overall positive feedback on the quality of the manuscript. We hope to have addressed all your concerns below. Also, we want to apologize for the extra time we needed to address the revision but the delivery of the kit to measure the adaptative immune response (Figure EV4) was delayed several times.

In the first paragraph of the results, the authors should describe the genome modification technologies used to knockout each of the four genes that they targeted. Relegating the information entirely to the supplemental compromises readability.

Done. Now the first section of the results is entitled "*Rational engineering of Mycoplasma pneumoniae as attenuated chassis*" (lines 174-193) in which we describe the obtention of the mutants by means of GP35 ssDNA recombinase using as substrates for recombination long stretches of ssDNA.

In the first paragraph of the results, the authors should describe how they validated that each gene was knocked out of each knockout strains.

We apologize, we checked by PCR the deletion of the target genes and the KO of each gene was further corroborated by the absence of the corresponding protein in a Mass Spectrometry analysis. As suggested this is now clearly specified in the first paragraph of the results (lines 174-193) and supplementary figures containing these data have been added (Figure EV1B and EV1C)

The authors report to characterize the phenotypes of their four KO strains by quantitative proteomics and cite Table S1 as the characterization. However, Table S1 is just a list of genes, numbers, and strains presented in undefined nomenclature. The authors report data as the mean of two technical replicates, but provide no information about the variability across replicates. Furthermore, they perform no analyses of changes in protein levels or whether those changes are statistically significant. They report in the main text that "Gene deletions were confirmed by the absence of the corresponding protein in the MS analysis". However, they do not define the units or scale they are using. What number equals absence of a protein? Given the poor presentation of Table S1 I cannot easily interpret whether or how any of the KO strains is different from the wild-type strain via proteomics. All of these issues should be corrected.

Apologies as the information included in Table S1 (now called DataSet EV1) has been clearly difficult to follow for all the reviewers. We made an effort to present it in a clearer way. In the first term, let us clarify that the information presented in the table is the mean of two technical replicates in all the cases except for Δ*mpn453* strain, for which only one technical replicate was available. As requested, for all the strains with two technical replicates a new column providing the Standard Deviation has been added to give information about the variability across replicates. Also, following your suggestion we assessed significant changes in protein abundance by independent two-sided t-test (scipy.stats python package v1.5.0 DOI:10.1038/s41592-019-0686-2), followed by multiple tests correction using Benjamini-Hochberg method with 5% family-wise false discovery rate. This analysis was restricted to those cases in which protein values from two technical replicates were available in all the strains, and no significant changes were found in the abundances of any protein compared to those observed in WT strain. This information is provided in the methods section (lines 827-838) Alternatively, to facilitate to the reader a qualitative measure of the main changes in the proteome of the different strains the average AUC values are gradually color-coded within each column from green (low abundance) to red (high abundance). See for instance *mpn134* that thanks to the color-coded scale it is easy to observe its upregulation in all the strains with *mpn133* deleted, although not enough to be statistically significant according to our analysis. Finally, we specified in the table legend that the units are the Area Under the Curve (AUC) for the average of the three best flying peptides which is one of the ways to do relative quantification of protein expression levels: (https://www.ncbi.nlm.nih.gov/pmc/articles/PMC3775642/). Regarding your question on what number equals absence of a protein, ND (Non-detected) would be equal to absence of a protein, but always having in mind that values close or below 10^7 are close to the detection

limit. This is also noted in the table legend. In few words unless several replicates are analyzed it is challenging to ensure a gene deletion just with proteomics data, but in this work it is used as a confirmation of the PCR analysis shown in Figure EV1B.

While Table S1 is a valuable resource to include with the paper, it is not a substitute for a simple figure showing the level of each target protein in the WT strain versus it's KO strain. Such a figure should be added.

Thanks for the suggestion, we have created a new figure (Figure EV1) that summarizes the generation and characterization of the engineered strains. Figure EV1A shows a scheme depicting the generation of long stretches of ssDNA that were later employed as substrate for recombination to generate the mutant strains. Figure EV1B shows the PCR screening done to confirm the editings carried out in all the strains. Figure EV1C includes the requested graph showing the expression level of each target protein in the different strains. Finally, Figure EV1D shows the doubling times of the different engineered strains.

There are similar issues with Table S2, which is being used to convey information about growth rate changes (or lack thereof) in their KO strains. Table S2 should be converted into a Figure to aid interpretation. All nomenclature should be defined in the legend.

We repeated the growth measurements for all the engineered strains (without taking data from previously published articles) by following the growth of the strains with three biological replicates and measuring protein biomass after 48 hours of growth. Following your suggestion and as stated above this information is now not only available in DataSet EV2, but also in Figure EV1D.

Why is it significant that no CV2 colonies were found in lung homogenate after delivery to mammary tissue? Were colonies of WT M. pneumoniae found in lung homogenate after delivery to mammary tissue? Absent that control, this experiment becomes weak/superfluous.

Sorry for not being clear enough in this regard. The experiment was done to show that the injected Mycoplasma strain did not distribute to other tissues. This is a control for biosafety. We did the same for the WT strain with the same results (now added to the text, lines 216-219).

The authors conclusion that P30 and GlpD are not major contributing factors to virulence in "mammary glands" should instead say "mouse mammary glands".

Done.

The units reported on the y-axes of Figure 2 are not defined, making the meaning of the changes in inflammatory mRNA values between WT and CT strains difficult to interpret. In the legend, the authors state "Data are the shown as mean +- DS of 2-DeltaCt*100 of at least 4 biological replicates for each sample". First, what is DS? Second, how is delta Ct calculated in this instance (what is the reference Ct value)? Why are different replicate numbers used in the different experiments? The number of replicates used should be listed for each sample.

This point has been raised by all the reviewers, apologies for not being clear. In the previous version the Ct method was applied using the following formula:

$$2^{-\Delta Ct} \times 100 = [2^{-(\text{Mean Ct gene unknown} - \text{Mean Ct gene housekeeping})}] \times 100$$

So basically, values were not normalized to the ones obtained in the PBS control group (for this reason PBS group was not always 1 and numbers seemed low). In anycase the reviewer is right that delta-delta CT method is more standard and allows easier comparison between groups. So in the new submission we employed the Ct method as follows:

$$\Delta Ct = \text{Mean Ct analyzed gene - Mean Ct } gapdH$$

$$\Delta\Delta Ct = \Delta Ct \text{ treated group - } \Delta Ct \text{ PBS group}$$

$$\text{Relative abundance} = 2^{-\Delta\Delta Ct}$$

Accordingly, we have changed the corresponding figures.

Regarding "DS" it was a typo on the previous submission, we wanted to say s.d. (standard deviation). Finally, regarding your concerns about the different numbers of replicates in the different experiments, it comes from the fact that no RNA extraction was performed for every single animal employed in the experiment and that sometimes it was tricky to load the multiwell plate for RTqPCR with all the samples we would have liked. Anyway, we believe that a minimal number of 4 replicates is high enough to trust the data. In anycase, following your advice for all the figures in which just means are represent (and not the individual values) we included in the figure legend the specific number of replicates per group (e.g. Figure 2, Figure EV1C, EV1D, EV2).

Overall the approach taken to discover secretion tags is innovative and yields exciting results. However, there are some issues with the presentation of the method and results that need to be cleared up. First, what the di-methyl labeling approach is and the rationale for why it was chosen should be described.

This is described in results now (lines 291-295):

By using a di-methyl labelling approach combined with MS (Tolonen et al, 2011) (see Methods), we studied the secretome at two different secretion time points (24 h and 72 h; two biological replicates). Essentially, we dimethyl–labelled (using heavy, medium or light isotopes) the proteins in the medium distinctly from the ones in the cells, and determined the relative abundance of a protein in both fractions using MS (see Methods).

In this way you can mix the supernatant and cell extracts (as described in methods lines 778-807), and distinguish the origin of the peptides (intracellular or extracellular) by means of the differential labeling.

Second, what is meant by 24h and 72h of growth? Hours of growth in a culture media after recovery from frozen? Do these numbers correspond to a growth phase or physiological state of the organism?

Sorry this was not explained well. This is now explained in Methods (lines 780-793):

For the secretome samples, the WT M129 strain was grown in Hayflick rich media for 3 days. The cultures were washed twice with PBS while attached, and twice after scraping and centrifugation at 14000 rpm. Cells were then split 1:10 into two different 150 cm2 flasks containing 40 mL of Hyprop media. Cells of both flasks were allowed to attach for 24 h and

then washed twice with PBS to remove all possible trace amounts from the horse serum coming from Hayflick media. After this, both flasks were filled again with 40 mL of Hyprop media and incubated at 37ºC for 72 h. Next, the culture supernatant and the cells of one the flasks were collected (representing the 72-h secretion time point); whereas the supernatant of the remaining flask was discarded, and the attached cells were washed twice with PBS and incubated for additional 24 h with 40 mL of fresh Hyprop media (representing the 24-h secretion time point) before being processed in the same way as the previous flask. For both flasks, cells were scraped in the same volume of Hyprop media as the collected supernatant. Cell suspensions were processed identically and in parallel to the supernatants, to avoid any bias from experimental procedure in the outcome.

Essentially, we are comparing 72 hours of secretion of proteins versus 24 hours. Taking into account how the samples were collected (with 24 h difference between them) and the fact that in Hyprop media *M. pneumoniae* grows really slowly the physiological state of the cells in these two conditions is quite similar.

Third, the fact that all the information describing Figure S3, an important figure in the paper, is somewhere else (Supplementary Results Section 1) makes it rather inconvenient to interpret Figure S3. I recommend converting Figure S3 to a proper main text figure and giving it a proper legend describing what was done.

Then I recommend moving the Section 1: Supplementary Results part to the methods. This will greatly increase readability.

Thanks for the suggestion, we have changed this figure to one of the main figures of the manuscript (Figure 3). According to the journal style, no supplementary results are allowed so we moved this section to methods.

The main text says 3-4 extra residues were added to the N-terminus of each secretion sequence, but the Table 1 legend says 5 such residues were added.

Apologies, regarding the number of extra residues added after the cleavage site there was a mistake in the previous version, and we apologize for it. We check the sequencing results of these constructs, and we can assure all of them carry 6 extra residues after the predicted cleavage site. The exact sequences that were tested can now be found in Dataset EV4

In Figure 3, what OD595 value corresponds to zero biofilm formation? A control needs to be added to indicate this value so that the effectiveness of the Dispersin B approach can be evaluated relative to a reference.

This value is totally neglectable (0.030) but it is true that it was important to include it as we did in figure 4A labeled as staining control (Note that former figure 3 is now figure 4)

In Figure 4B-D, to what extent does the catheter stain purple/absorb at 595nm if no biofilm is ever added to it? This information is important to understand the efficacy of the Mycoplasma method and a new control should be added showing it.

Also neglectable values, we plotted the values in figures 5C and 5D and also added a picture of a non infected catheter stained with crystal violet in Figure 5B to illustrate this issue. (note that former Figure 4 is now Figure 5)

The "sequences of interest" in Table S5 do not do an effective job conveying the complete sequence of the engineered plasmids, which will make it more difficult for future researchers to follow up on this work work. The complete annotated sequences of all plasmids should be uploaded to Genbank and the accession #s given.

We made an effort to provide all the information of interest in DataSet EV6. In addition, we have uploaded the complete sequence of the most relevant plasmids of this study (i.e., those designed to secrete Dispersin B and Lysostaphin, as well as those employed as template for the generation of ssDNA recombineering substrates required for the generation of the mutant strains) to the European Nucleotide Archive (ENA). The accession number is PRJEB45050

1st Revision - Editorial Decision 15th Jul 2021

Thank you for sending us your revised manuscript. We have now heard back from the two reviewers who agreed to evaluate your study. The reviewers are satisfied with the modifications made and they are supportive of publication.

Before we formally accept the study for publication, we would ask you to address some minor editorial issues listed below.

REFEREE REPORTS

Reviewer #1:

The authors have done a good job in addressing the points raised during the original round of reviews. We recommend the revised paper for publication in MSB.

Reviewer #2:

The revised manuscript submitted by Garrido and colleagues has been significantly improved. The story is more clearly presented in this version and details that were missing or difficult to understand have been clarified. My concerns from the previous round of review have been adequately addressed.

2nd Authors' Response to Reviewers 14th Sep 2021

The authors have made all requested editorial changes.

ACCEPTED **15th Sep 2021**

Thank you again for sending us your revised manuscript. We are now satisfied with the modifications made and I am pleased to inform you that your paper has been accepted for publication.

EMBO PRESS

YOU MUST COMPLETE ALL CELLS WITH A PINK BACKGROUND ↓

PLEASE NOTE THAT THIS CHECKLIST WILL BE PUBLISHED ALONGSIDE YOUR PAPER

Corresponding Author Name: María Lluch-Senar; María-Jesús Grilló; Luis Serrano
Journal Submitted to: Molecular Systems Biology
Manuscript Number: MSB-2020-10145

Reporting Checklist For Life Sciences Articles (Rev. June 2017)

This checklist is used to ensure good reporting standards and to improve the reproducibility of published results. These guidelines are consistent with the Principles and Guidelines for Reporting Preclinical Research issued by the NIH in 2014. Please follow the journal's authorship guidelines in preparing your manuscript.

A- Figures

1. Data

The data shown in figures should satisfy the following conditions:

- the data were obtained and processed according to the field's best practice and are presented to reflect the results of the experiments in an accurate and unbiased manner.
- figure panels include only data points, measurements or observations that can be compared to each other in a scientifically meaningful way.
- graphs include clearly labeled error bars for independent experiments and sample sizes. Unless justified, error bars should not be shown for technical replicates.
- if n< 5, the individual data points from each experiment should be plotted and any statistical test employed should be justified
- Source Data should be included to report the data underlying graphs. Please follow the guidelines set out in the author ship guidelines on Data Presentation.

2. Captions

Each figure caption should contain the following information, for each panel where they are relevant:

- a specification of the experimental system investigated (eg cell line, species name).
- the assay(s) and method(s) used to carry out the reported observations and measurements
- an explicit mention of the biological and chemical entity(ies) that are being measured.
- an explicit mention of the biological and chemical entity(ies) that are altered/varied/perturbed in a controlled manner.

- the exact sample size (n) for each experimental group/condition, given as a number, not a range;
- a description of the sample collection allowing the reader to understand whether the samples represent technical or biological replicates (including how many animals, litters, cultures, etc.).
- a statement of how many times the experiment shown was independently replicated in the laboratory.
- definitions of statistical methods and measures:
  - common tests, such as t-test (please specify whether paired vs. unpaired), simple χ2 tests, Wilcoxon and Mann-Whitney tests, can be unambiguously identified by name only, but more complex techniques should be described in the methods section;
  - are tests one-sided or two-sided?
  - are there adjustments for multiple comparisons?
  - exact statistical test results, e.g., P values = x but not P values < x;
  - definition of 'center values' as median or average;
  - definition of error bars as s.d. or s.e.m.

Any descriptions too long for the figure legend should be included in the methods section and/or with the source data.

In the pink boxes below, please ensure that the answers to the following questions are reported in the manuscript itself. Every question should be answered. If the question is not relevant to your research, please write NA (non applicable). We encourage you to include a specific subsection in the methods section for statistics, reagents, animal models and human subjects.

USEFUL LINKS FOR COMPLETING THIS FORM

http://www.antibodypedia.com
http://1degreebio.org
http://www.equator-network.org/reporting-guidelines/improving-bioscience-research-repor
http://grants.nih.gov/grants/olaw/olaw.htm
http://www.mrc.ac.uk/Ourresearch/Ethicsresearchguidance/Useofanimals/index.htm
http://ClinicalTrials.gov
http://www.consort-statement.org
http://www.consort-statement.org/checklists/view/32-consort/66-title
http://www.equator-network.org/reporting-guidelines/reporting-recommendations-for-tumo
http://datadryad.org
http://figshare.com
http://www.ncbi.nlm.nih.gov/gap
http://www.ebi.ac.uk/ega
http://biomodels.net/
http://biomodels.net/miriam/
http://jjj.biochem.sun.ac.za
http://oba.od.nih.gov/biosecurity/biosecurity_documents.html
http://www.selectagents.gov/

B- Statistics and general methods

Please fill out these boxes ↓ (Do not worry if you cannot see all your text once you press return)

1.a. How was the sample size chosen to ensure adequate power to detect a pre-specified effect size?	The sample size was chosen regarding the existing scientific literature for each type of experiment
1.b. For animal studies, include a statement about sample size estimate even if no statistical methods were used.	The sample size was chosen regarding the existing scientific literature for each type of experiment
2. Describe inclusion/exclusion criteria if samples or animals were excluded from the analysis. Were the criteria pre-established?	All the samples and animals processed were included in the analysis
3. Were any steps taken to minimize the effects of subjective bias when allocating animals/samples to treatment (e.g. randomization procedure)? If yes, please describe.	To to ensure that each experimental unit has an equal probability of receiving a particular treatment, all mice were randomly allocated in cages before starting the experiment and, thereafter, mice of each cage were processed together. Moreover, blind treatments were generally administered in mice experiments.
For animal studies, include a statement about randomization even if no randomization was used.	All our studies were performed under simple randomization procedures.
4.a. Were any steps taken to minimize the effects of subjective bias during group allocation or/and when assessing results (e.g. blinding of the investigator)? If yes please describe.	All the experiments were performed minimising the possibilities of bias. Mice were radomly allocated in cages with food and water ad libitum before starting the experiment; treatments administered to mice were blind administered by the technician, and identify by codes for traceability; raw data were also processed blind and codified for traceability.
4.b. For animal studies, include a statement about blinding even if no blinding was done	All the experiments were performed minimising the possibilities of bias. Mice were radomly allocated in cages with food and water ad libitum before starting the experiment; treatments administered to mice were blind administered by the technician, and identify by codes for traceability; raw data were also processed blind and codified for traceability.
5. For every figure, are statistical tests justified as appropriate?	The statistical tests were evaluated by an ethical and animal experiments committee, supervised by the "Órgano Habilitado" of CSIC (Spain) and approved by the competent authority on animal experiments, according to the current legislation regional, national and European rules.
Do the data meet the assumptions of the tests (e.g., normal distribution)? Describe any methods used to assess it.	Yes, we assessed that all data accomplished the assumptions required by the statistical tests; sistematically, we normalised the raw data obtained in order to warranty their normalisation before their statistical treatment.
Is there an estimate of variation within each group of data?	The ANOVA test considers the intra-group variance of the results obtained.

Is the variance similar between the groups that are being statistically compared?	Yes, the ANOVA test we made provided the output parameter of intragroup variance, thus, we assessed that.

C- Reagents

6. To show that antibodies were profiled for use in the system under study (assay and species), provide a citation, catalog number and/or clone number, supplementary information or reference to an antibody validation profile. e.g., Antibodypedia (see link list at top right), 1DegreeBio (see link list at top right).	NA
7. Identify the source of cell lines and report if they were recently authenticated (e.g., by STR profiling) and tested for mycoplasma contamination.	NA

* for all hyperlinks, please see the table at the top right of the document

D- Animal Models

8. Report species, strain, gender, age of animals and genetic modification status where applicable. Please detail housing and husbandry conditions and the source of animals.	Mice (Mus musculus) CD1 females older than 6 weeks, outbreed (no genetically modified). All animals were purchased at at Charles River Laboratories (France) aging 4-week-old and housed in appropriate cages with water and food ad libitum, under the pathogen-free conditions at the Institute of Agrobiotechnology (authorization code ES/31-2016-000002-CR-SU-US). For mammary gland infections, pregnancies were synchronized by dark–light circadian cycles, males were housed with the females for 3 days, and the offspring were removed at day 10 of lactation. For catheter experiments, mice were used after 2 weeks of housing. Animal handling and procedures were performed following the FELASA and ARRIVE guidelines (Kilkenny et al, 2010), in accordance with the current European (Directive 86/609/EEC) and National (Real Decreto 53/2013) legislations, and with the approval of the competent authority of Navarra Government (Resoluciones 281/2020 and 282/2020).
9. For experiments involving live vertebrates, include a statement of compliance with ethical regulations and identify the committee(s) approving the experiments.	Animal handling and procedures were performed following the FELASA and ARRIVE guidelines (Kilkenny et al, 2010), in accordance with the current European (Directive 86/609/EEC) and National (Real Decreto 53/2013) legislations, and with the approval of the competent authority of Navarra Government (Resoluciones 281/2020 and 282/2020).
10. We recommend consulting the ARRIVE guidelines (see link list at top right) (PLoS Biol. 8(6), e1000412, 2010) to ensure that other relevant aspects of animal studies are adequately reported. See author guidelines, under 'Reporting Guidelines'. See also: NIH (see link list at top right) and MRC (see link list at top right) recommendations. Please confirm compliance.	We confirm the compliance of this point

E- Human Subjects

11. Identify the committee(s) approving the study protocol.	NA
12. Include a statement confirming that informed consent was obtained from all subjects and that the experiments conformed to the principles set out in the WMA Declaration of Helsinki and the Department of Health and Human Services Belmont Report.	NA
13. For publication of patient photos, include a statement confirming that consent to publish was obtained.	NA
14. Report any restrictions on the availability (and/or on the use) of human data or samples.	NA
15. Report the clinical trial registration number (at ClinicalTrials.gov or equivalent), where applicable.	NA
16. For phase II and III randomized controlled trials, please refer to the CONSORT flow diagram (see link list at top right) and submit the CONSORT checklist (see link list at top right) with your submission. See author guidelines, under 'Reporting Guidelines'. Please confirm you have submitted this list.	NA
17. For tumor marker prognostic studies, we recommend that you follow the REMARK reporting guidelines (see link list at top right). See author guidelines, under 'Reporting Guidelines'. Please confirm you have followed these guidelines.	NA

F- Data Accessibility

18: Provide a "Data Availability" section at the end of the Materials & Methods, listing the accession codes for data generated in this study and deposited in a public database (e.g. RNA-Seq data: Gene Expression Omnibus GSE39462, Proteomics data: PRIDE PXD000208 etc.) Please refer to our author guidelines for 'Data Deposition'.<br><br>Data deposition in a public repository is mandatory for:<br>a. Protein, DNA and RNA sequences<br>b. Macromolecular structures<br>c. Crystallographic data for small molecules<br>d. Functional genomics data<br>e. Proteomics and molecular interactions	Raw data of mass spectroscopy spectra is deposited at PRIDE database with the submission reference: 1-20201104-61226 and the Submission Path: /nfs/pride/drop/pride-drop-003/samuel.miravet_20201104_161411. The complete sequence of the most relevant plasmids designed in this study as well as the genome assembly of CV2 strain can be found at the European Nucleotide Archive (ENA) under the study accession number PRJEB45050
19. Deposition is strongly recommended for any datasets that are central and integral to the study; please consider the journal's data policy. If no structured public repository exists for a given data type, we encourage the provision of datasets in the manuscript as a Supplementary Document (see author guidelines under 'Expanded View' or in unstructured repositories such as Dryad (see link list at top right) or Figshare (see link list at top right).	We have deposited the datasets in public databases
20. Access to human clinical and genomic datasets should be provided with as few restrictions as possible while respecting ethical obligations to the patients and relevant medical and legal issues. If practically possible and compatible with the individual consent agreement used in the study, such data should be deposited in one of the major public access-controlled repositories such as dbGAP (see link list at top right) or EGA (see link list at top right).	NA
21. Computational models that are central and integral to a study should be shared without restrictions and provided in a machine-readable form. The relevant accession numbers or links should be provided. When possible, standardized format (SBML, CellML) should be used instead of scripts (e.g. MATLAB). Authors are strongly encouraged to follow the MIRIAM guidelines (see link list at top right) and deposit their model in a public database such as Biomodels (see link list at top right) or JWS Online (see link list at top right). If computer source code is provided with the paper, it should be deposited in a public repository or included in supplementary information.	NA

G- Dual use research of concern

22. Could your study fall under dual use research restrictions? Please check biosecurity documents (see link list at top right) and list of select agents and toxins (APHIS/CDC) (see link list at top right). According to our biosecurity guidelines, provide a statement only if it could.	The experiments performed with Mycoplasma pneumoniae and Staphylococcus aureus were supervised by the Biosecurity Committees of the Public University of Navarre and CSIC (for experiments at the Institute of Agrobiotechnology - IdAB) or the CRG (for experiments at CRG). Experiments with Mycoplasma GMOs were performed at the authorised BSL2 facilities of the CRG and IdAB-CSIC (A/ES/12/I-33 at CRG and code A/ES/10/I-77 at IdAB) and with GMOs authorisations provided by the Spanish National Biosecurity Commission (CNB codes: A/ES/12/37 at CRG and A/ES/16/77 at IdAB).
